# Integrating climate policies in the sustainability analysis of green chemicals†

**DOI:** 10.1039/d4gc00392f

**Published:** 2024-04-18

**Authors:** Abhinandan Nabera, Antonio José Martín, Robert Istrate, Javier Pérez-Ramírez, Gonzalo Guillén-Gosálbez

**Affiliations:** a Institute for Chemical and Bioengineering, Department of Chemistry and Applied Biosciences, ETH Zürich Vladimir Prelog Weg 1 Zürich 8093 Switzerland jpr@chem.ethz.ch gonzalo.guillen.gosalbez@chem.ethz.ch; b Institute of Environmental Sciences (CML), Leiden University Einsteinweg 2 2333 CC Leiden The Netherlands

## Abstract

New and enhanced processes will not be the only drivers toward a sustainable chemical industry. Implementing climate policies will impact all components of the chemical supply chain over the following decades, making improvements in energy generation, material extraction, or transportation contribute to reducing the overall impacts of chemical technologies. Including this synergistic effect when comparing technologies offers a clearer vision of their future potential and may allow researchers to support their sustainability propositions more strongly. Ammonia and methanol production account for more than fifty percent of the CO_2_ emissions in this industry and are, therefore, excellent case studies. This work performs a prospective life cycle assessment until 2050 for fossil, blue, wind, and solar-based technologies under climate policies aiming to limit the global temperature rise to 1.5 °C, 2 °C, or 3.5 °C. The first finding is the inability of fossil-based routes to reduce their CO_2_ emissions beyond 10% by 2050 without tailored decarbonisation strategies, regardless of the chemical and climate policy considered. In contrast, green routes may produce chemicals with around 90% fewer emissions than today and even with net negative emissions (on a cradle-to-gate basis), as in the case of methanol (up to −1.4 kg CO_2_-eq per kg), mainly due to the contributions of technology development and increasing penetration of renewable energies. Overall, the combined production of these chemicals could be net-zero by 2050 despite their predicted two to fivefold increase in demand. Lastly, we propose a roadmap for progressive implementation by 2050 of green routes in 26 regions worldwide, applying the criterion of at least 80% reduction in climate change impacts when compared to their fossil alternatives. Furthermore, an exploratory prospective techno-economic assessment showed that by 2050, green routes could become more economically attractive. This work offers quantitative arguments to reinforce research, development, and policymaking efforts on green chemical routes reliant on renewable energies.

## Abstract 2

The grand transition towards green pathways for chemical production, alongside ambitious climate policies, could achieve 90% reduction in climate change impacts by 2050, while fossil routes offer a reduction of 10% in the best case.

## Graphical abstract

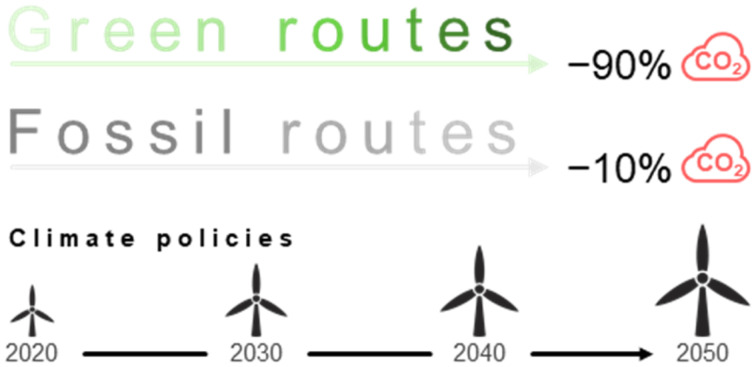

## Introduction

The carbon emissions of the chemical industry are hard to abate due to its heavy reliance on oil and natural gas, resulting in the annual release of 5.6 Gt CO_2_-eq (10% of global anthropogenic GHG emissions).^1^ However, only one-third of these emissions are direct emissions (scope 1), while the rest are indirect emissions linked to energy generation (scope 2) and other activities in the chemical supply chain (scope 3).^1^ Consequently, decarbonisation trends in the economy, mostly motivated by climate policies resulting from the Paris Agreement,^2–4^ particularly in energy generation, are expected to decrease the chemical industry's environmental footprint substantially. Nonetheless, very often, current environmental assessments of chemicals partially address future changes concerning scope 2 emissions, yet leave scope 3 emissions at their current levels, potentially leading to more pessimistic estimates.

Platform chemicals, such as ammonia and methanol, are key to reducing GHG emissions due to their significant production volumes,^5–7^ which are forecasted to at least double over the next decades.^8–11^ They are both produced following well-established catalytic fossil routes, based first on steam methane reforming (SMR)^12^ to generate the hydrogen required by the Haber–Bosch process (ammonia)^13,14^ and syngas for CO hydrogenation (methanol).^15,16^ However, the ongoing trend is to shift to non-fossil alternative routes. Specifically, green hydrogen can be generated through water electrolysis, using renewable electricity sources such as wind or solar.^12,17^ The existing Haber–Bosch process could then use this green hydrogen as the input^17^ or even rely on other low-carbon hydrogen sources, *e.g.*, biomass gasification.^7,18^ Moreover, green methanol could be produced using the reverse water–gas shift reaction, transforming captured CO_2_ into CO and also utilising low-carbon hydrogen.^6^ The strong reliance of these routes on low-carbon electricity to produce hydrogen suggests that they could benefit substantially from the increasing penetration of renewable energy in the future electricity mix,^19,20^ yet the specific extent to which they will improve remains unknown.

The standardised life cycle assessment (LCA) methodology^21,22^ has been extensively applied to assess and compare the environmental performance of fossil and green production routes for hydrogen,^23,24^ ammonia,^25,26^ and methanol.^27–29^ These analyses have consistently highlighted the potential environmental superiority of the green routes over their fossil counterparts. However, the majority of these LCA studies have not yet considered the temporal evolution of impacts linked to expected changes in industrial sectors.^30,31^ Pioneering prospective studies have evaluated the effect of future technologies on chemical production. However, only changes in the power mixes supplying energy to the chemical plant (foreground system) were often considered.^32^ Very recently, prospective studies covering diverse modifications in the background system (all the supply chain activities connected to an industrial system) have started to emerge in the literature,^33,34^ yet applications to chemicals are still very scarce.^35^

In this work, we assess the cradle-to-gate environmental impacts of fossil and green ammonia, and methanol production from 2020 to 2050, including expected changes in the power, materials, and transportation sectors under three climate policies aiming to limit global warming below 3.5 °C, 2 °C, and 1.5 °C by 2100, respectively. We show the inability of the business-as-usual fossil routes to decrease their emissions significantly in these scenarios, and the potential of green routes to decrease emissions by 90%, even resulting in negative emissions (on a cradle-to-gate basis) in the case of methanol. We estimate that decarbonising the power sector will contribute to 60% of the impact decrease on average. Lastly, a roadmap for the worldwide implementation of green routes by 2050 proposes a pathway to introduce these routes while minimising overall impacts.

Furthermore, in this work, we streamline messages to overcome the terminology barrier that often hinders effective communication while working on catalysts, reactors, and process scales. Our conclusions strongly support research activities related to these areas.

## Methods

We conduct an attributional prospective LCA of all the production technologies under consideration, following the four distinct phases outlined in ISO 14040 and 14044 standards.^21,22^ Our analysis focuses on the functional unit, defined as the production of 1 kg of a specific chemical (hydrogen, ammonia, or methanol) using a particular production technology. We employ a cradle-to-gate approach, encompassing all relevant inputs and outputs from both the technosphere (*e.g.*, economic flows like electricity) and biosphere (*e.g.*, elementary flows like CO_2_ emissions and natural resources) necessary for chemical production. In the context of a prospective LCA, we utilise the *premise* v1.5.8 ^36^ framework to construct future background data. The life cycle impact assessment (LCIA) is performed utilising the IPCC 2013 ^37^ 100-year average global warming potentials (GWPs) to evaluate climate change impacts, and the Environmental Footprint 3.0 ^38^ (EF) methods to quantify 14 other impact categories. We quantify both the global average and region-specific impacts by considering the variability in power mixes across regions. For detailed insights into each phase of our assessment, please refer to section 1 of the ESI.†

## Results and discussion

### Climate policies will drastically reduce the carbon footprint of green chemicals

**Fig. 1:**
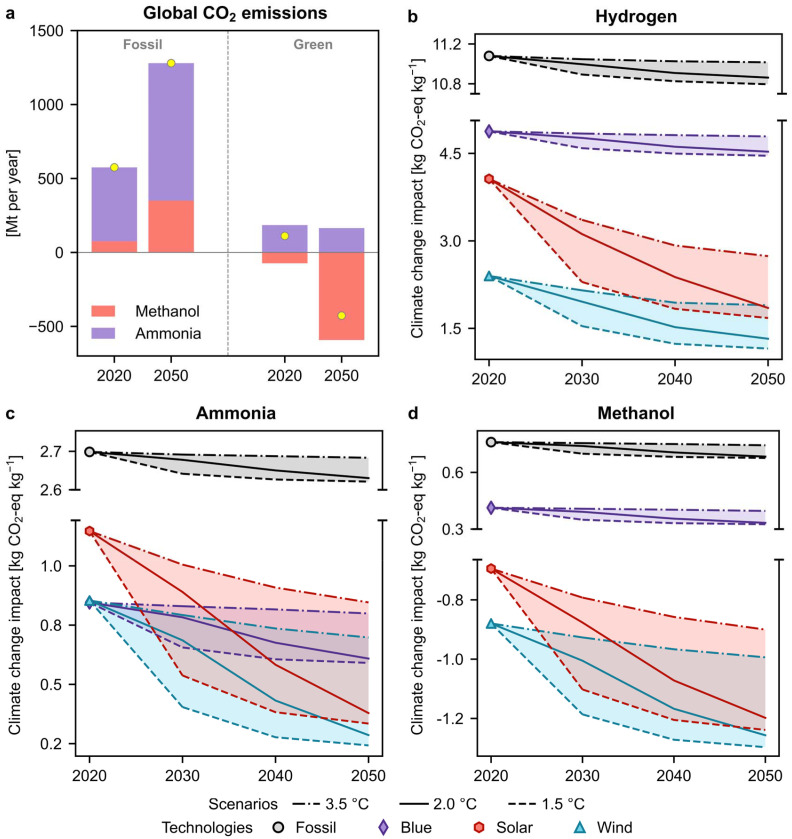
(a) Global CO_2_ emissions for ammonia and methanol in 2020 and 2050. The emissions were calculated based on the average impacts of fossil or green (solar and wind-based) routes, as determined through prospective LCAs. Values are available in Table S2.† (b)–(d) Global 100-year average cradle-to-gate climate change impacts for fossil, blue, and green (both solar- and wind-based) production of (b) hydrogen, (c) ammonia, and (d) methanol from 2020 to 2050 in three climate policy scenarios. Lower impacts are observed under the influence of more ambitious policies (2 °C and 1.5 °C scenarios), which can be attributed to the decarbonised production of electricity.

Fig. 1a presents a summary of the current situation and the overall outcome of this prospective study. In 2020, the combined demand for ammonia and methanol amounted to 285 Mt, resulting in CO_2_ emissions as high as 575 Mt. Projecting to 2050, with our continued reliance on fossil fuels, it is anticipated that the combined demand will reach approximately 855 Mt,^8–11^ leading to potential CO_2_ emissions of up to 1280 Mt. This work concludes that meeting this demand with net-zero emissions, or even achieving net negative performance depending on the scenario, is feasible by adopting decarbonised routes such as wind and solar, as illustrated in the figure. We find that utilising green routes (assuming an average of solar and wind energy sources) for the production of ammonia and methanol would have led to emissions as high as 112 Mt in 2020. However, in 2050, both these chemicals combined could result in net negative emissions of around 427 Mt (on a cradle-to-gate basis). The origin of these results are explained in detail below.

Fig. 1b–d displays the global average temporal evolution of impacts for both chemicals, considering all scenarios and technologies our model considers, as well as the hydrogen essential for their production. The climate change impacts of green routes (solar and wind) for the three chemicals are expected to decrease by an average of 26% over time, even in the baseline 3.5 °C scenario. Compared to current emissions (fossil scenario in 2020), green routes could produce hydrogen and ammonia in the 1.5 °C scenario with 90% fewer emissions and could even result in net negative CO_2_ emissions in the case of methanol (*ca*. −1.4 kg CO_2_-eq per kg, on a cradle-to-gate basis). The negative emissions are a result of considering atmospheric CO_2_, which would otherwise contribute to atmospheric GHG levels, being captured and contained in methanol. However, fossil routes would benefit much less from future decarbonisation trends, showing modest carbon footprint reductions below 10%, even in the best case. We observe that the impacts of fossil-based ammonia production pathways in this study align well with the literature estimates for the 3.5 °C scenario.^34^

**Fig. 2:**
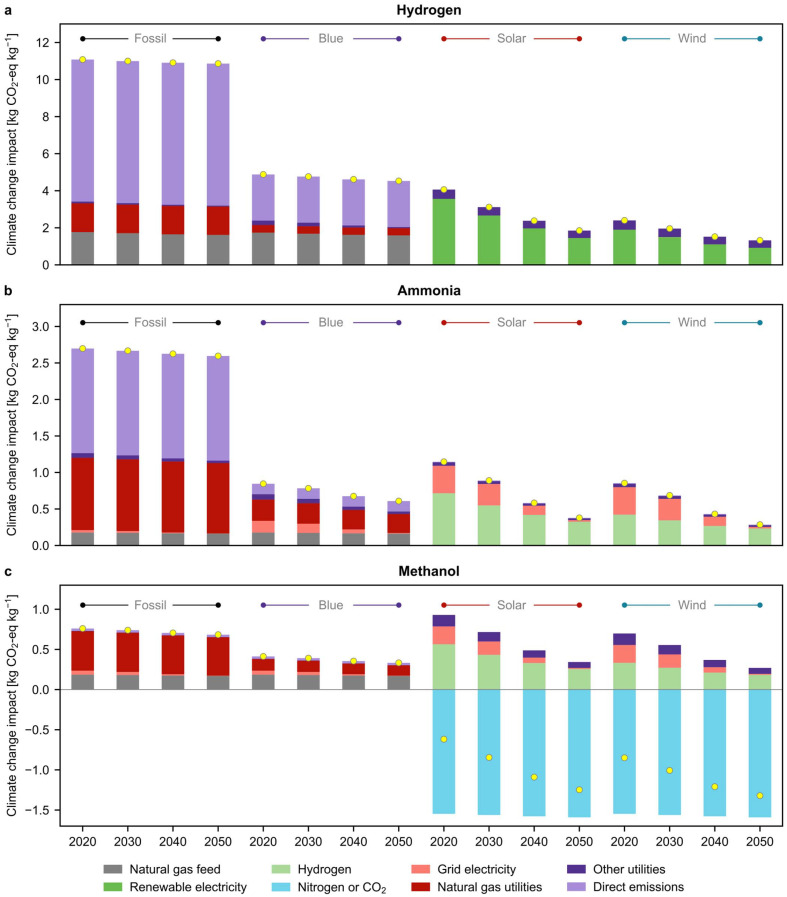
Breakdown of projections for global 100-year average cradle-to-gate climate change impacts for fossil, blue, and green (both solar- and wind-based) (a) hydrogen, (b) ammonia, and (c) methanol. The results presented here are for the scenario compatible with limiting global warming to below 2 °C. The yellow dots represent the net impact of the specified technology. Corresponding analyses for the 3.5 °C and 1.5 °C scenarios can be found in Fig. S1 and S2,† respectively. ‘Natural gas feed’ refers to the carbon emissions associated with the natural gas used as a feedstock. On the other hand, ‘Natural gas utilities’ refer to the emissions associated with the natural gas used for heating, including combustion emissions. ‘Nitrogen’ is utilised for the production of green ammonia, while ‘CO_2_’ is utilised for the production of green methanol, respectively. Standalone evolution plots considering decarbonisation in only cement and steel, electricity, transport, or technological advancements are shown in Fig. S3–S6.†

Fig. 2 delves into the reasons underlying the temporal patterns for the 2 °C scenario (results for the 3.5 °C and 1.5 °C scenarios can be found in Fig. S1 and S2 of the ESI,† showing similar trends), focusing on the role played by the decarbonisation of the inputs to the chemical plant. For both wind and solar-based green hydrogen, the core of the reduction lies in decreased consumption (*via* electrolyser efficiency gains) and further decarbonisation of renewable electricity. Notably, the decarbonisation of the power sector results in substantial impact reductions, leading to an average 64% reduction in climate change impacts (Fig. S3†), while anticipated improvements in electrolyser efficiencies and other technological advancements are projected to result in only 9% reduction in impacts (Fig. S4†) for these green routes. Besides energy efficiency improvements in water electrolysers (from 61% in 2020 to 73% in 2050),^39^ the solar based route will benefit from improved module efficiency of multi-Si PV panels, projected to increase from 17% to 24% in accordance with the *premise* framework.^36^

On the other hand, it is expected that the carbon footprint of onshore wind electricity will moderately decline by 2050. This decline is primarily attributed to decarbonisation initiatives across various sectors, *i.e.*, power (Fig. S3†), materials (cement and steel, Fig. S5†), and transportation (Fig. S6†), rather than efficiency improvements in wind turbines (Fig. S4†). Regarding the power sector, solar-based routes greatly improve their performance, while wind-based pathways lead to lower environmental gains due to the negligible evolution of load factors and efficiencies for onshore wind turbines considered in *premise*.^36^ This aligns with investigations indicating that wind turbine performances are plateauing, so significant reductions in carbon intensity should be alternatively attained *via* manufacturing and end-of-life recycling of turbines.^40,41^ Overall, in the context of climate change mitigation, solar technologies are projected to play a pivotal role in the chemical industry by 2050.

Since green ammonia and methanol routes (Fig. 2b and c) are based on the consumption of green hydrogen, environmental benefits in the latter are directly reflected in their reduced emissions due to the large hydrogen use (light green bars). In addition, green ammonia and methanol routes will also benefit from the 18-fold reduction in carbon emissions linked to the decarbonisation of future electricity mixes (light red), required to power the plant compressors. Notably, green methanol exhibits overall negative impacts on a cradle-to-gate basis, primarily due to the CO_2_ captured from the atmosphere and utilised as feedstock, as highlighted before.

A radically different evolution is predicted for fossil routes, blue hydrogen (Fig. 2a), and methanol (Fig. 2c). These pathways benefit much less from future decarbonisation efforts than their green analogues because carbon emissions primarily originate from direct emissions (CO_2_ removed from the syngas stream in the SMR process in hydrogen and ammonia production, and purged stream in methanol synthesis), followed by emissions linked to natural gas used as feedstock and heating utility. Furthermore, our results indicate that solar- and wind-based routes, despite currently displaying larger carbon footprints than blue ammonia, are projected to outperform blue ammonia in all future scenarios, revealing that considering temporal dynamics can lead to a new ranking of technologies.^7^ We note that blue routes could be considered as an interim solution but not fully sustainable in the long term due to their reliance on carbon storage, whose global capacity is limited.^42^ Additionally, CCS would reduce the footprint of fossil methanol much less than in the case of fossil ammonia since, in the former, natural gas (whose production contributes to climate change) provides both the carbon and hydrogen sources. Lastly, electrification of heating could also contribute to emission reduction in the fossil routes,^43^ yet it would not be sufficient to outperform the green routes unless CCS is deployed in tandem. Moreover, it is important to acknowledge that the lower barriers to renewable energy adoption for power generation in the 1.5 °C and 2 °C scenarios, compared to the 3.5 °C scenario, also play a crucial role in shaping outcomes.

Previous works have assessed the economic viability of green chemicals amidst increased energy prices due to Europe's energy crisis.^44^ In this work, we expand this analysis to include exploratory prospective evaluations of ammonia and methanol production costs (see section 7 and Fig. S7 of the ESI†). We find that green routes may become economically more attractive by 2050, especially considering the decrease in green hydrogen production costs.^45^ Solar-based production routes may demonstrate the highest reduction in climate change impacts over time, approaching the performance levels of their corresponding wind-based production, again nuancing previous studies that have consistently found wind-based routes to outperform solar pathways.^6,7^

### Burden shifting and convergence of green routes

**Fig. 3:**
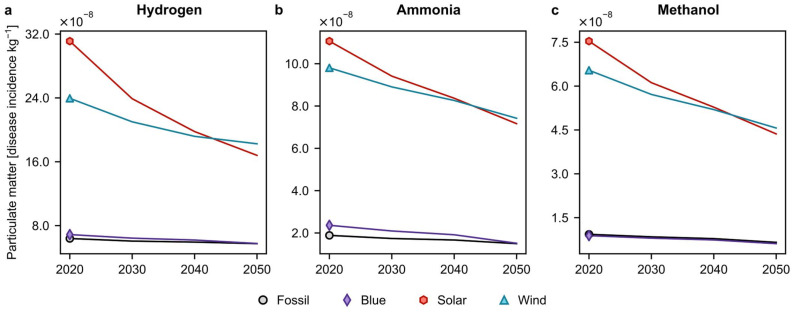
Global average cradle-to-gate particulate matter impacts of fossil, blue, and green (both solar- and wind-based) production of (a) hydrogen, (b) ammonia, and (c) methanol from 2020 to 2050. This impact category was selected based on its quality level, following the recommendations of the European Commission's Joint Research Centre. The results reported are for the scenario compatible with limiting global warming below 2 °C. Similar results for all quality level I impact categories other than climate change, namely ozone depletion and particulate matter formation, in the 3.5 °C and 1.5 °C scenarios are displayed in Fig. S8.† A breakdown analysis for the particulate matter formation and ozone depletion categories in the 2 °C scenario is shown in Fig. S9 and S10,† respectively. Other environmental impact categories, according to the environmental footprint (EF) v3.0 method, considering all scenarios, are available in Fig. S11† for hydrogen, Fig. S12† for ammonia, and Fig. S13† for methanol, respectively.

We next extend our analysis beyond the carbon footprint to assess the potential risk of burden shifting, *i.e.*, the worsening of other impacts when attempting to combat climate change. Here, we focus on particulate matter, which is the most interesting case among those defined as level I (recommended and satisfactory) by the European Commission's Joint Research Centre.^46^ This category serves as an indicator of the likelihood of disease occurrence related to particulate matter formation. Moreover, certain gases in the atmosphere, such as sulfur dioxide and nitric oxide, can react with organic volatile compounds to form particulate matter or act as catalysts for their formation.^47^ These particles can reflect sunlight, thereby reducing the solar radiation reaching the Earth's surface and lowering the temperature. Despite this cooling effect (which is anticipated to be limited), particulate matter formation has consequences for human health, causing respiratory diseases, thus underscoring the importance of assessing impacts other than climate change. Fig. 3 presents the global average evolution of this impact for all technologies and chemicals in the 2 °C scenario (other scenarios show similar trends, Fig. S8†).

Consistent with other works,^35,48^ green routes exhibit larger particulate matter impacts than their fossil counterparts, indicating the occurrence of burden-shifting, yet the environmental gap shrinks over time. The breakdown analysis in Fig. S9† identifies renewable electricity utilised for hydrogen production as the primary source of particulate matter impact for green routes originating from the construction of solar panels and wind turbines. Another interesting observation is the gradual convergence of impacts among green routes, suggesting, again, a change in the ranking from wind to solar in the vicinity of 2040, mirroring the trends seen in climate change. Fig. S9† shows the faster evolution of solar technology towards higher efficiency as the reason behind this shift.

Furthermore, in contrast to climate change, the fossil and blue routes display overall low impacts with potential for reductions of up to 35%, particularly in ammonia and methanol production. These substantial reductions can be attributed to decreased impacts from the mixes powering the compressors (Fig. S9†). These findings underscore the environmental trade-offs that often arise when combating climate change.^49^

Ozone depletion, another category considered as level I and indicating airborne pollutants responsible for depleting the stratospheric ozone layer, exhibits, however, a much more similar behaviour to climate change (Fig. S8†). Green routes outperform fossil ones and show a positive trend toward reducing impacts, propelled by the decrease in renewable energy needs over time (Fig. S10†). Equivalent analyses for 12 other impact categories, categorised as quality levels II and III, are presented in Fig. S11† for hydrogen, Fig. S12† for ammonia, and Fig. S13† for methanol. They generally reveal a noticeable temporal decrease in impacts for green routes, not mirrored by fossil routes, and the occurrence of burden shifting in some cases, which tends to become less severe over time.

Overall, these results further emphasise that the sustainability assessment of these production routes should include different impact categories besides climate change to make well-informed decisions.

### A strategy for the implementation of green routes until 2050

One relevant corollary of the previous sections is that the rate of decrease in climate change impacts over the next decades of green routes will largely depend on their location. This follows from the diverse power mixes deployed worldwide, found to be the main driving force behind this trend (Fig. S3†). In order to maximise global benefits, an effective strategy may involve favouring their implementation in regions where impacts below a defined threshold are achieved. This would lead to a progressively larger number of appealing regions as the decarbonisation of power generation advances. In view of the results presented in Fig. 1b–d, the target of 80% impact reduction compared to the corresponding fossil-based scenarios in 2020 was established for green hydrogen and ammonia, yielding thresholds of 8.9 and 2.2 kg CO_2_-eq per kg, respectively. For the case of green methanol, with already negative climate change impacts, the criterion was set at 80% reduction compared to the impact of the solar route in 2020 (−1.1 kg CO_2_-eq per kg).

**Fig. 4:**
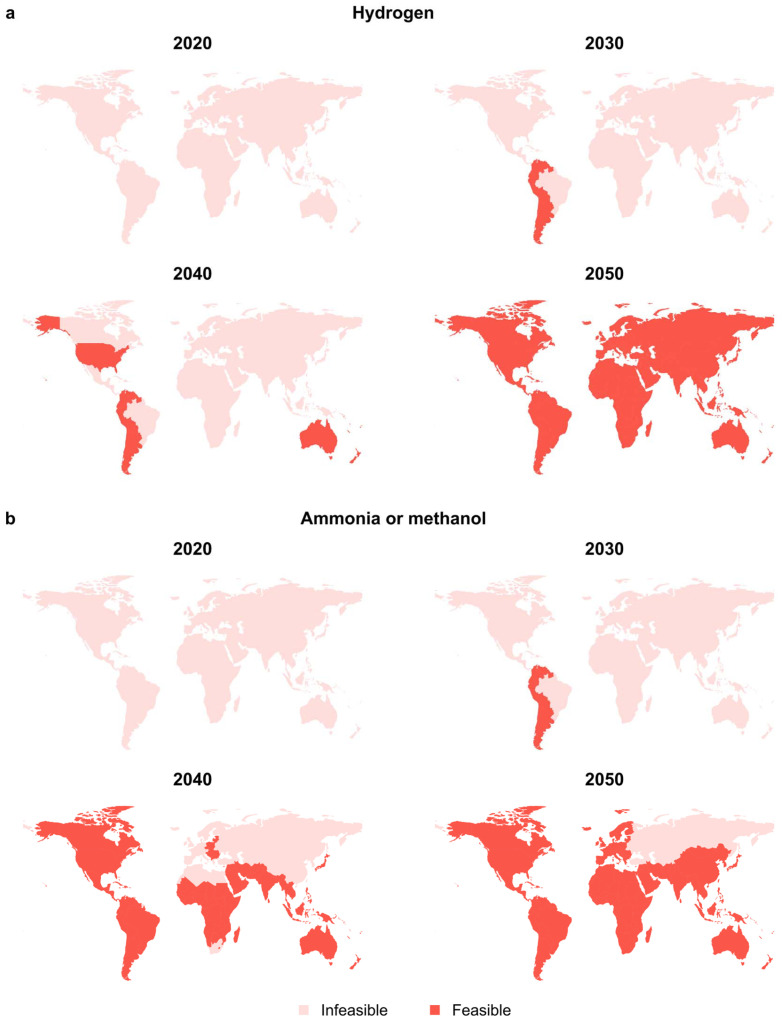
Regional assessment of the cradle-to-gate climate change impacts for solar-based production routes of (a) hydrogen and (b) ammonia or methanol. The highlighted regions in red for a specific year indicate locations with impacts lower than a defined threshold, considering scenarios compatible with limiting global warming to below 2 °C. For hydrogen and ammonia, thresholds of 80% reduction from their respective fossil-based impacts in 2020 (8.9 and 2.2 kg CO_2_ per kg, respectively) are considered. Meanwhile, for methanol, an 80% reduction from its respective solar-based impacts in 2020 (−1.1 kg CO_2_-eq per kg) is utilised due to its net negative impacts. Corresponding analyses for the wind-based routes are presented in Fig. S14–S16,† whereas the analyses for the 3.5 °C and 1.5 °C scenarios are available in Fig. S17–S24,† respectively. Furthermore, the relative change in climate change impacts from 2020 to 2050 for the green routes in all the scenarios are displayed in Fig. S25–S30.†

We next performed a temporal regional assessment across 26 regions worldwide in the 1.5 °C, 2 °C, and 3.5 °C scenarios to suggest a roadmap for each chemical–green route–policy trio. As an illustrative case, Fig. 4 displays the regional assessment for solar-based routes in the 2 °C scenario for the three chemicals. Results for wind-based routes can be found in Fig. S14–16 of the ESI,† showing similar trends. Furthermore, analyses corresponding to the 3.5 °C and 1.5 °C scenarios are presented in Fig. S17 and S18 for solar and in Fig. S19–S24† for wind-based production, respectively.

In Fig. 4a, the case of hydrogen suggests that the first region able to meet this stringent criterion will be South America, excluding Brazil, by 2030, primarily because their electricity mix is not yet decarbonised. The United States of America and Australia are projected to achieve these targets by 2040, and virtually all regions in the world will be suitable one decade later when energy decarbonisation efforts will be mostly completed in this scenario. For other extreme scenarios, like the 3.5 °C case, the analysis predicts that only South American countries (again except Brazil) would be able to achieve the required performance by 2050, whereas the strongest environmental commitment brought by the 1.5 °C case would make all regions worldwide suitable by 2040.

In Fig. 4b, we displayed the results for both ammonia and methanol, as they yield identical results in this analysis. It is expected that by 2030, regions suitable for the production of low carbon solar-based ammonia and methanol will match those of hydrogen. However, as we move towards 2040, a greater number of regions are projected to attain the specified targets compared to hydrogen. Furthermore, by 2050, every region is expected to meet the established goals, with the exception of Russia, Turkey, and Central Asia. Similarly, for the hydrogen case, adopting the 3.5 °C policy would render all regions unable to meet the specified target until 2040, when the same South American countries identified for hydrogen would join and remain the only recommended locations until 2050. Following the 1.5 °C option would make most of the world eligible already by 2030 except Turkey and Central Asia.

A complementary analysis compares the relative changes for a fixed location in the climate change impacts in all climate scenarios (Fig. S25–S30†). Those locations with the largest relative variations are thus predicted to experience a deeper structural change in their supply chains and will require more extensive capital investments. In the 2 °C scenario and paying attention to solar-based routes (Fig. S26†), ammonia displays potential for significant relative impact reduction in 2050, with the lowest reduction observed in Turkey and the highest achieved in Western Africa. In contrast, hydrogen shows a much more homogeneous decrease rate. Similarly, solar-based methanol production exhibits an impact reduction ranging from around 50% in Canada to more than 200% in South Africa. The wind-based routes (Fig. S29†) show similar trends to the corresponding solar scenarios.

Overall, this analysis reveals which regions worldwide will first evolve in different scenarios to achieve low carbon production of platform chemicals, enabling effective long-term strategic planning and policy making. In addition, it reinforces the relevance of committing to ambitious climate policies. Low carbon platform chemicals may be produced in most regions worldwide within the next two decades, bringing an encouraging message fostering research and development on green chemical production with spatial granularity.

## Conclusions and outlook

This work emphasises that transitioning to green pathways for the production of chemicals can significantly reduce climate change impacts, particularly under ambitious climate policies. Policies limiting global warming to 3.5 °C, 2 °C, or 1.5 °C were studied herein. Together with technological advancements, energy decarbonisation pathways embedded in climate policies will lead, on their own, to environmental benefits of approximately 60% reductions in impact on average because of the strong interconnection of chemical production with other elements of the supply chain. Overall, by 2050, hydrogen and ammonia could be produced with approximately 90% fewer emissions than today, whereas methanol could even lead to net negative emissions (on a cradle-to-gate basis). In contrast, fossil routes without any tailored decarbonisation strategies will achieve only up to approximately 10% reductions in impacts, rendering them unsuitable options towards a sustainable chemical industry.

Comprehensive analysis beyond climate change is needed for informed decision making to detect and quantify burden shifting, as illustrated by the case of the particulate matter category. Finally, the variable evolution of electricity generation mixes worldwide has allowed the construction of a regional roadmap. It describes locations that will progressively display the potential to produce chemicals with 80% reductions in emissions compared to 2020, with the South- and North American regions showing the earliest feasibility between 2030 and 2040.

Beyond the examples of thermocatalytic routes analysed here, these results demonstrate the general appeal of green routes, with an emphasis on those with a strong reliance on electricity, like electrocatalytic ones. The methodology derived herein, and the distilled insights provide tools for researchers, policymakers, industry leaders, and investors to encourage the green production of chemicals. However, conclusions reached at the system process level do not always reach other communities, primarily due to the inherent complexity of such analyses and segregated communication channels. We hope our study will permeate to offer resolute support to activities in these fields.

## Author contributions

A. N.: conceptualisation, methodology, visualisation, formal analysis, writing – original draft, and writing – review and editing; A. M.: conceptualisation, methodology, visualisation, writing – original draft, and writing – review and editing; R. I.: conceptualisation, methodology, validation, writing – original draft, and writing – review and editing; J. P. R.: conceptualisation, writing – review and editing, supervision, and project administration; G. G. G.: conceptualisation, validation, writing – original draft, writing – review and editing, supervision, and project administration.

## Conflicts of interest

There are no conflicts to declare.

## Supplementary Material

GC-026-D4GC00392F-s001
