# Supplementary material for: Integrating climate policies in the sustainability analysis of green chemicals
Source: Green Chem. 2024 Apr 18;26(11):6461–9. doi: 10.1039/d4gc00392f (PMC11148852; doi:10.1039/d4gc00392f)
Supplement: GC-026-D4GC00392F-s001 [file GC-026-D4GC00392F-s001.pdf]

Electronic Supplementary Material (ESI) for Green Chemistry.
This journal is © The Royal Society of Chemistry 2024

Electronic Supplementary Information

Integrating climate policies in the sustainability analysis of green chemicals

Abhinandan Nabera,[a] Antonio José Martín,[a] Robert Istrate,[b] Javier Pérez-Ramírez*[a] and Gonzalo Guillén-Gosálbez*[b]

[a]Institute for Chemical and Bioengineering, Department of Chemistry and Applied Biosciences, ETH Zürich, Vladimir-Prelog-Weg 1, Zürich 8093, Switzerland.

[b]Institute of Environmental Sciences (CML), Leiden University, Einsteinweg 2, 2333 CC Leiden, The Netherlands.

Table of contents

1. Prospective life cycle assessment methodology 3
2. Prospective life cycle inventories 5
3. Assumptions and limitations of the study 7
4. Global demand and emissions in 2020 and 2050 8
5. Breakdown of climate change impacts under the 3.5 °C and 1.5 °C scenarios 9
6. Sector wise evolution of climate change impacts 11
7. Prospective techno-economic assessment 13
8. Ozone depletion and particulate matter formation impacts under all scenarios 16
9. Breakdown of particulate matter and ozone depletion under the 2 °C scenario 17
10. Additional impact categories under the 2 °C scenario 19
11. Additional regional implementation roadmaps 23

1. Prospective life cycle assessment methodology

1.1. Production technologies considered

We investigate the environmental performance of alternative production routes for hydrogen, ammonia, and methanol. For hydrogen, we analyse four typical sources: grey hydrogen from steam methane reforming (SMR), blue hydrogen from SMR coupled with carbon capture and storage (CCS), and green hydrogen from water electrolysis using solar photovoltaic (PV) or onshore wind electricity. We focus only on solar PV and onshore wind due to their scalability, making them favourable options in comparison to other renewable power generation technologies. Our selection of these technologies for green hydrogen production aligns with the projections and expectations outlined in the context of the hydrogen economy.[1,2] Similarly, we compare four alternative production routes for ammonia *via* the Haber-Bosch process: conventional synthesis from natural gas, synthesis from natural gas coupled with CCS (blue), and synthesis from solar or wind-based electrolytic hydrogen (green). In the case of methanol, we evaluate similar pathways to ammonia production, except for the green routes, where, in addition to green hydrogen, we utilise CO_2 captured from the atmosphere as a feedstock.

1.2. Life cycle assessment

We conduct an attributional prospective LCA of all the production technologies under consideration, following the four distinct phases outlined in ISO 14040 and 14044 standards.[3,4] In the following sections, we provide detailed insights into each phase of our assessment.

1.2.1. Phase 1: Goal and scope definition

In this work, we consider the functional unit as the production of 1 kg of a chemical (hydrogen, ammonia, or methanol) *via* a specific production technology. Our analysis takes a cradle-to-gate approach, encompassing all relevant upstream inputs and outputs from the technosphere (*i.e.*, economic flows such as electricity) and biosphere (*i.e.*, elementary flows such as CO_2 emissions and natural resources) required to produce the chemical. We chose this approach to avoid making assumptions about chemical usage, which would introduce uncertainties into our analysis.[5] Moreover, the use and end-of-life stages would be the same in alternative production routes. We assess environmental impacts spanning from 2020 to 2050, considering future scenarios for three climate targets (specifically, 3.5 °C, 2 °C, and 1.5 °C). Additionally, we quantify both the global average impact and region-specific impacts by considering regional variability in terms of all technosphere flows, *e.g.*, electricity mixes. We select the most suitable datasets that match the regional scope of our analysis. In cases where data for a particular country or region is not accessible, we instead rely on global or rest of the world inventories.

1.2.2. Phase 2: Inventory analysis

Herein, we provide a brief overview of the references used to derive the life cycle inventories (LCIs) employed in our analysis. The LCIs for fossil, blue, and green hydrogen in this study were automatically generated for all future scenarios using the *premise* v1.5.8[6] framework from Ecoinvent v3.8.[7] Specifically, the LCIs for fossil and blue hydrogen were based on Antonini *et al.*[8], while those for hydrogen from electrolysis were derived from Bareiß *et al.*[9]. Similarly, fossil ammonia was derived from the existing inventories in Ecoinvent.[7] On the other hand, for blue ammonia, the fossil inventories were modified to account for CCS (**Section 2**), and green ammonia inventories were derived from D'Angelo *et al.*[10] For fossil and blue methanol, a similar procedure to that used for ammonia was adopted. For green methanol, *premise*-generated inventories adopted from Hank *et al.*[11] were used in this analysis.

In general, the aim of this study is to evaluate the environmental performance associated with crucial platform chemicals at various stages of technological advancement and decarbonisation of key sectors. To accommodate different future scenarios, we need to modify the inventory data to reflect technological and socio-economic changes

expected in the coming years. Besides adjusting technical parameters within primary processes (*e.g.*, efficiencies), background processes should be updated accordingly (*e.g.*, to reflect an increase of renewables in electricity mixes). In this regard, we utilise the future economic projections derived from Integrated Assessment Models (IAMs).

IAMs, such as IMAGE (Integrated Model to Assess the Global Environment), provide a comprehensive understanding of the interlinked relationships among society, the biosphere, and the climate system.[12] In this regard, we utilise the *premise* v1.5.8[6] framework to construct future background data that aligns with the results derived from the IMAGE model. The IMAGE model assumes particular climate change mitigation scenarios and Shared Socio-economic Pathways (SSPs) that describe the relationship between challenges for mitigation and adoption. Therefore, in our study, we assume SSP2 ("middle-of-the-road"). For the mitigation scenarios, we consider three Representative Concentration Pathways (RCPs) with respect to their goal to limit global mean surface temperature (GMST) as follows:

Baseline scenario (RCP6), limiting to 3.5 °C (SSP2-Base from *premise*).
Moderate scenario (RCP2.6), limiting to 2 °C (SSP2-RCP26 from *premise*).
Ambitious scenario (RCP1.9), limiting to 1.5 °C (SSP2-RCP19 from *premise*).

Moreover, future power generation scenarios derived from IMAGE also consider the anticipated increase in power demand, as well as the expansion of the existing transmission grid to accommodate the evolving energy landscape. All calculations were performed using the Brightway2 framework.[13] The initial data used in this study for all the chemicals was generated automatically based on *premise* v1.5.8,[6] sourced from Ecoinvent v3.8.[7] A more comprehensive overview of the life cycle inventory data for each production pathway of a chemical across all scenarios is briefly explained in **Section 2**. It should be noted that the predictions arising from our analysis and discussed herein must be understood as the most likely trend based on extending currently available knowledge. The lack of information about background processes is an inherent feature of prospective LCA, which hampers the inclusion of uncertainty in our analysis. A broader discussion on the limitations and assumptions of this work is available in **Section 3**.

1.2.3. Phase 3: Impact assessment methods

This phase encompasses the life cycle impact assessment (LCIA), where multiple impact categories are assessed to unearth potential hidden impacts and trade-offs. We employ the IPCC 2013[14] global warming potentials (GWPs) to quantify the climate change impacts and the Environmental Footprint 3.0[15] (EF) methods to quantify 14 other impact categories. Here, we concentrate on climate change, particulate matter formation, and ozone depletion categories. These categories were chosen following the recommendations of the European Commission's Joint Research Centre, with a specific focus on quality level I, which is considered recommended and satisfactory.[16] The results for 12 other categories, which belong to quality levels II and III,[16] are briefly described in **Section 10** of the ESI.

1.2.4. Phase 4: Interpretation

In this assessment, we calculate the relative change in impacts for 26 specific regions, as defined by the IMAGE model, from 2020 to 2050. The following equation is used to derive the results:

$$\%\ \text{change} = \frac{\mathrm{CCI}_{2050,\,i} - \mathrm{CCI}_{2020,\,i}}{\mathrm{CCI}_{2020,\,i}} \tag{1}$$

where '*i*' represents the specific region, as defined by the IMAGE model.

2. Prospective life cycle inventories

The life cycle inventories (LCIs) employed for various production routes of hydrogen, ammonia, and methanol are briefly described in this section. For all the production routes, four distinct sets of inventories were created, encompassing the worldwide economy in 2020, as well as inventories reflecting the global market in 2030, 2040, and 2050, with decarbonisation scenarios following 3.5 °C, 2 °C, and 1.5 °C pathways. Detailed information regarding changes in background processes in each year, such as improvements in solar panel efficiencies, advancements in steel production technologies, and decarbonisation of the electricity mix, performed through *premise* v1.5.8[6] can be found in the documentation. Additionally, for hydrogen production from water electrolysis (green hydrogen routes), electrolyser efficiencies and lifetimes for each respective year can be found in the documentation, in accordance with the references cited for their respective LCIs in the following sections.

2.1. Hydrogen

The study analysed various routes for hydrogen production, including grey or fossil hydrogen from steam methane reforming (SMR), blue hydrogen from SMR coupled with carbon capture and storage (CCS), and green hydrogen from water electrolysis using solar photovoltaic (PV) or onshore wind energy. The life cycle inventories utilised and adapted for grey, blue, and green hydrogen in this study were generated automatically through *premise*,[6] employing data from Ecoinvent v3.8.[7] These inventories were originally extracted from Antonini *et al*.[8] for fossil and blue hydrogen at 25 bar from SMR and Bareiß *et al*.[9] for green hydrogen at 30 bar. Notably, in the hydrogen from electrolysis inventories that are already modelled with grid electricity, the electricity flows were replaced with 'electricity production, wind, >3 MW turbine, onshore' and 'electricity production, photovoltaic, 570 kWp open ground installation, multi-Si' for hydrogen from wind and solar power, respectively. This substitution assumes that electrolytic hydrogen production will primarily rely on renewable energy sources rather than conventional grid electricity.

2.2. Ammonia

For ammonia, this study focused on four different production routes *via* the Haber-Bosch process, including conventional synthesis from natural gas (fossil), synthesis from natural gas coupled with CCS (blue), and synthesis from solar or wind-based electrolytic hydrogen (green). For fossil ammonia production, we utilised existing inventories from Ecoinvent v3.8,[7] specifically 'ammonia production, steam reforming, liquid'. Similarly, for blue ammonia, we modified the respective fossil ammonia inventories to account for CCS. We capture, compress, and store CO_2 from both syngas and the flue gas resulting from the combustion of natural gas in the SMR process.[10] The CO_2 is assumed to be captured through a mature technology, *i.e.*, monoethanolamine-based chemical absorption.[17] After compression to 110 bar, the captured CO_2 is transported through a 200 km pipeline to a geological site, where it is injected to a depth of 1000 m.[17] The CCS inventories, which involve capturing CO_2 through monoethanolamine-based chemical absorption, were extracted from Volkart *et al*.[17]. Furthermore, the energy flows for transportation and injection were extracted from the work done by Antonini *et al*.[8]. Green ammonia inventories utilising solar and wind-based hydrogen from water electrolysis were based on the work done by D'Angelo *et al*.,[10] substituting the respective hydrogen flows with solar or wind-based hydrogen described earlier.

2.3. Methanol

In this study, we examined various methanol production routes, focusing on four specific routes: conventional synthesis from natural gas (fossil), synthesis from natural gas with CCS (blue), synthesis from captured CO_2 and solar or wind-based electrolytic hydrogen (green). For fossil methanol production, we referenced existing data from Ecoinvent v3.8,[7] specifically the 'methanol production' inventory. Similarly, for blue methanol, we adjusted the fossil methanol inventories to incorporate CCS. Since emissions from methanol synthesis are minimal to non-existent, CCS is only applied to emissions from the combustion of natural gas for heating in the case of blue methanol. Green methanol inventories, utilising hydrogen generated from the electrolysis of water, powered by solar or wind energy, were based

on the work of Hank *et al.*[11]. In these inventories, we substituted the respective hydrogen sources with solar or wind-based hydrogen, as previously explained. Notably, the green routes for methanol production also utilise CO_2 as a feedstock. Therefore, in this study, we assume that the CO_2 is captured from the atmosphere employing a solvent-based Direct Air Capture (DAC) technology. The heat source for this technology was considered to be heat pumps with a COP of 2.9. The current and prospective inventories for this technology were based on the work done by Qiu *et al.*,[18] further substituting the grid electricity flows with onshore wind electricity, assuming a renewable power source for the operation of the capture unit.

Table S1 Brief overview of prospective life cycle inventories utilised in this work.

Chemical	Technology	Source	Activity (*premise* or Ecoinvent)
Hydrogen	Fossil – SMR	*premise* based on Antonini *et al.*[8]	hydrogen production, steam methane reforming of natural gas, 25 bar
	Blue – SMR coupled with CCS	*premise* based on Antonini *et al.*[8]	hydrogen production, steam methane reforming of natural gas, with CCS (MDEA, 98% eff.), 25 bar
	Solar powered water electrolysis	*premise* based on Bareiß *et al.*[9] (substituting grid electricity with 'electricity production, photovoltaic, 570 kWp open ground installation, multi-Si')	hydrogen production, gaseous, 30 bar, from PEM electrolysis, from grid electricity (substituting grid with solar)
	Wind powered water electrolysis	*premise* based on Bareiß *et al.*[9] (substituting grid electricity with 'electricity production, wind, >3 MW turbine, onshore')	hydrogen production, gaseous, 30 bar, from PEM electrolysis, from grid electricity (substituting grid with onshore wind)
Ammonia	Fossil – SMR	Ecoinvent v3.8 ('ammonia production, steam reforming, liquid')	ammonia production, steam reforming, liquid
	Blue – SMR coupled with CCS	Ecoinvent v3.8 ('ammonia production, steam reforming, liquid') coupled with CCS using monoethanolamine-based chemical absorption inventory extracted from Volkart *et al.*[17]	ammonia production, steam reforming, liquid (CCS inventory from Volkart *et al.*[17] for both syngas and flue gas)
	Solar	D'Angelo *et al.*[10] (substituting with solar-based hydrogen)	Inventory from D'Angelo *et al.*[10] using solar-based hydrogen
	Wind	D'Angelo *et al.*[10] (substituting with wind-based hydrogen)	Inventory from D'Angelo *et al.*[10] using wind-based hydrogen
Methanol	Fossil – SMR	Ecoinvent v3.8 ('methanol production')	methanol production
	Blue – SMR coupled with CCS	Ecoinvent v3.8 ('methanol production') coupled with CCS using monoethanolamine-based chemical absorption inventory extracted from Volkart *et al.*[17]	methanol production (CCS inventory from Volkart *et al.*[17] for flue gas)
	Solar	Hank *et al.*[11] (using carbon dioxide from DAC sourced from *premise* based on Qiu *et al.*[18] and solar-based hydrogen)	methanol synthesis, hydrogen from electrolysis, CO_2 from DAC (using solar-based hydrogen)
	Wind	Hank *et al.*[11] (using carbon dioxide from DAC sourced from *premise* based on Qiu *et al.*[18] and wind-based hydrogen)	methanol synthesis, hydrogen from electrolysis, CO_2 from DAC (using wind-based hydrogen)

3. Assumptions and limitations of the study

- The findings in this study are influenced by the assumptions of the IMAGE IAM, which generated the background inventories. Our study utilises the *premise*[6] python package to generate prospective background inventories.
- We sourced inventory data from Ecoinvent v3.8, utilising a cut-off system model.[7] We selected the most representative datasets in alignment with the regional scope of our analysis. When the data for a specific country or region was unavailable, we utilised the global or rest of the world-based inventories instead.
- The life cycle assessment employing various impact assessment methods is calculated for the production of 1 kg of the product, *i.e.*, hydrogen, ammonia, or methanol. A cradle-to-gate assessment is carried out for all categories, avoiding assumptions about the use phase of these platform chemicals, thereby reducing uncertainties.
- We assume only multi-Si PV panels for solar technologies in our analysis. An improvement in efficiency is considered for these technologies. However, for onshore wind turbines, no improvement in efficiency is considered in the automatically generated and utilised inventories. Nonetheless, data trends suggest that the majority of reductions in carbon intensity will stem from the production and recycling of wind turbines at the end of their life cycle. Therefore, it is projected that there will be insignificant improvements in load factors and efficiencies.[19]
- In our analysis of all production technologies, we assume continuous operation of compressors and pumps, and are thus powered by grid electricity. In contrast, we assume that the water electrolysis process is powered using onshore wind or solar electricity, and the DAC unit is exclusively powered employing onshore wind electricity. Our study does not account for any potential energy storage requirements for both solar and wind electricity. Furthermore, we allocate all environmental burdens to hydrogen, as the current market cannot absorb the excess oxygen generated as a by-product of water electrolysis.[10]
- For green hydrogen production, we exclusively considered proton exchange membrane (PEM) water electrolysis powered by solar or onshore wind electricity due to its high potential for large-scale deployment.
- Conducting a prospective life cycle assessment (LCA) based on predicted data derived from Integrated Assessment Models (IAMs) has its limitations. First, *premise* is currently focussed on power generation, cement and steel production, transport, and fuels. Second, the outcomes in the IAM scenario are constrained on the assumptions employed within the IAM framework, and these assumptions may or may not accurately reflect the potential trajectories of the global economy.
- Uncertainties are not considered in this analysis as it requires extensive information about diverse background processes and the inventories generated with *premise* lack sufficient data points.
- In our study, which adopts a prospective LCA, we have addressed potential impacts from increased mining, yet acknowledge limitations in considering future changes due to insufficient data on advancements in the mining industry.

4. Global demand and emissions in 2020 and 2050

Table S2 Global demand and emissions of hydrogen, ammonia, and methanol in 2020 and 2050. The emissions in 2050 are calculated assuming the average impacts of solar and wind-based routes to display a best-case scenario.

	**Year**	**Ammonia**	**Methanol**
**Demand**	2020	185	100
	2050	355	500
**Fossil emissions***	2020	499	76
	2050	929	351
**Green emissions***	2020	185	-73
	2050	165	-592

*Considering the anticipated average climate change impacts per kg of fossil or green (solar- or wind-based) chemical production across different climate policies (displayed in **Fig. 1**).

5. Breakdown of climate change impacts under the 3.5 °C and 1.5 °C scenarios

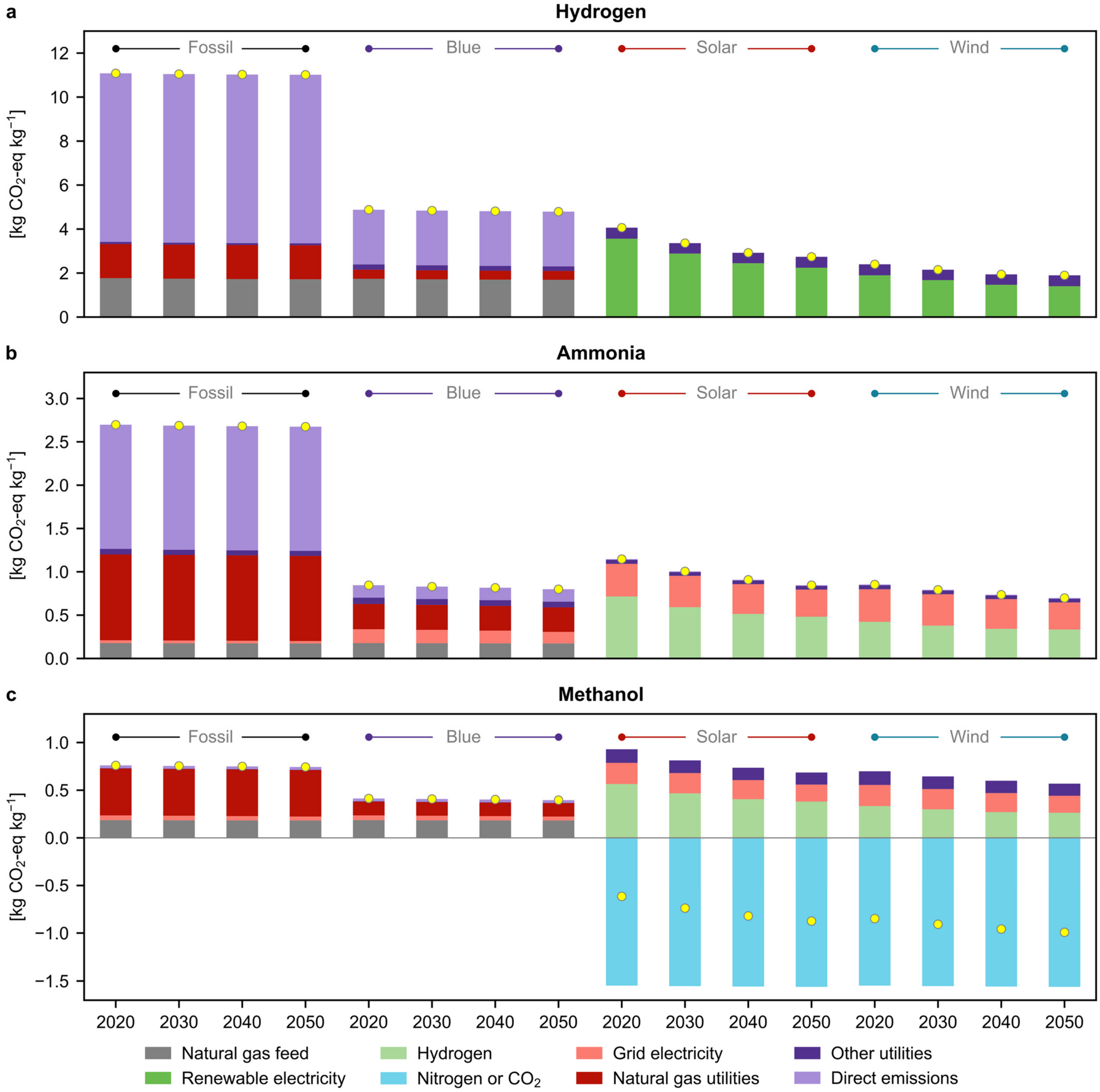

Fig. S1 Breakdown projections of global average cradle-to-gate climate change impacts for fossil, blue, and green (both solar- and wind-based) **(a)** hydrogen, **(b)** ammonia, and **(c)** methanol. The results reported are for the scenario compatible with limiting global warming below 3.5 °C. The yellow dots represent the net impact of the specified technology. In general, the projections indicate that there will only be marginal improvements.

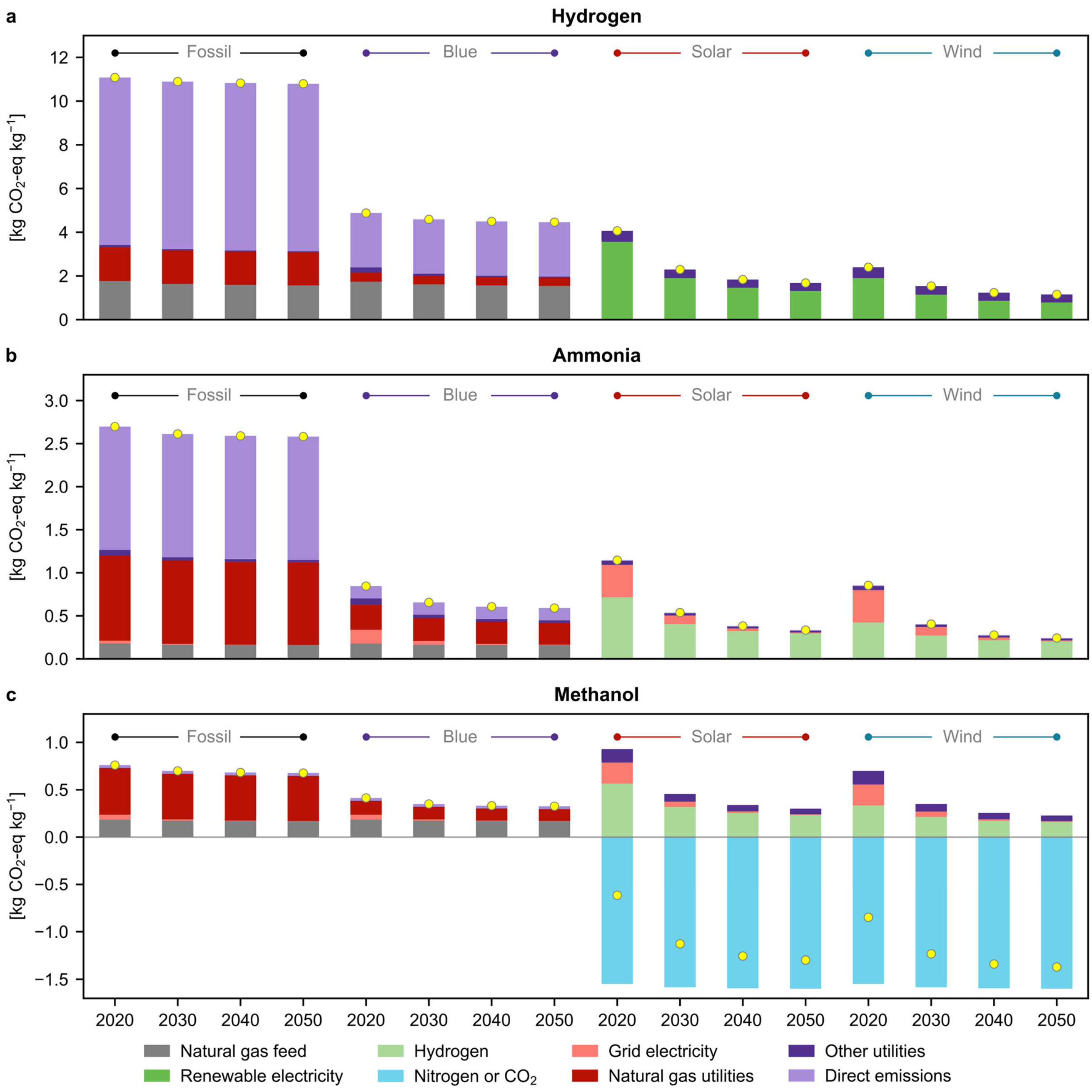

Fig. S2 Breakdown projections of global average cradle-to-gate climate change impacts for fossil, blue, and green (both solar- and wind-based) **(a)** hydrogen, **(b)** ammonia, and **(c)** methanol. The results reported are for the scenario compatible with limiting global warming below 1.5 °C. The yellow dots represent the net impact of the specified technology.

6. Sector wise evolution of climate change impacts

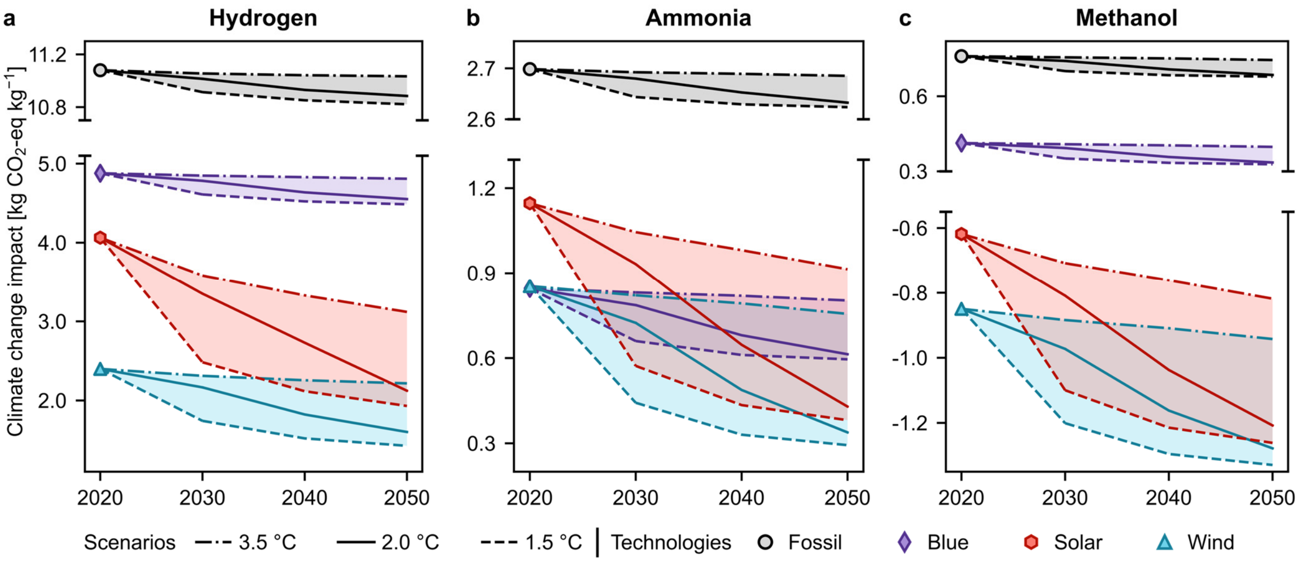

Fig. S3 Global average cradle-to-gate climate change impacts for fossil, blue, and green (both solar- and wind-based) production of **(a)** hydrogen, **(b)** ammonia, and **(c)** methanol from 2020 to 2050 under three climate policy scenarios, considering decarbonisation only in the power sector.

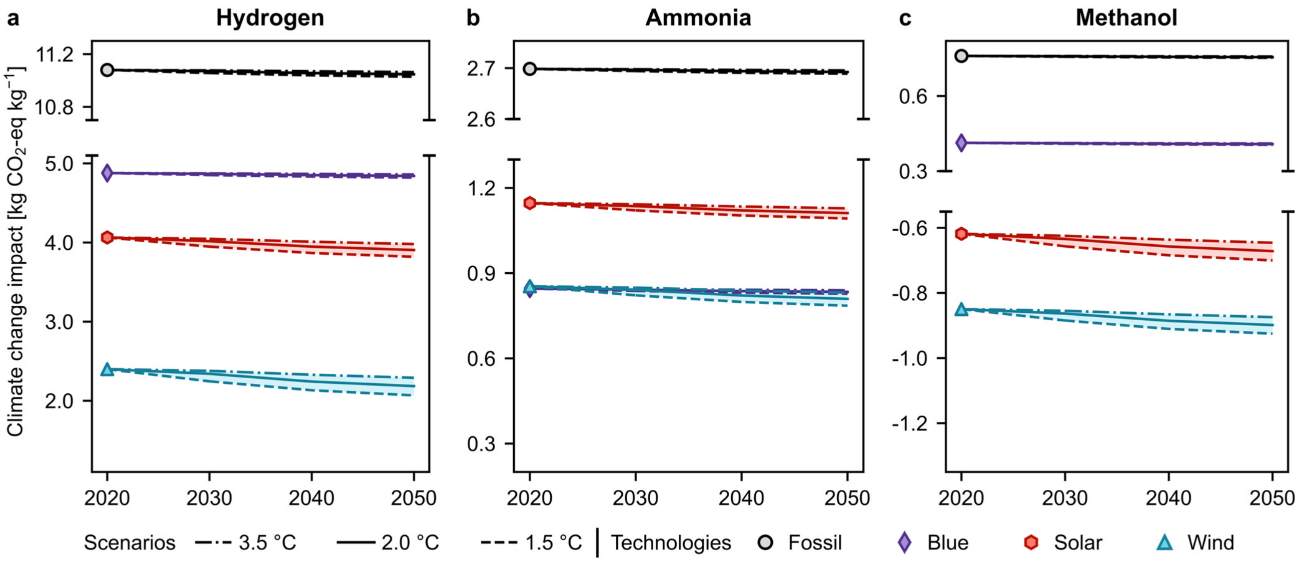

Fig. S4 Global average cradle-to-gate climate change impacts for fossil, blue, and green (both solar- and wind-based) production of **(a)** hydrogen, **(b)** ammonia, and **(c)** methanol from 2020 to 2050 under three climate policy scenarios, considering only technological advancements.

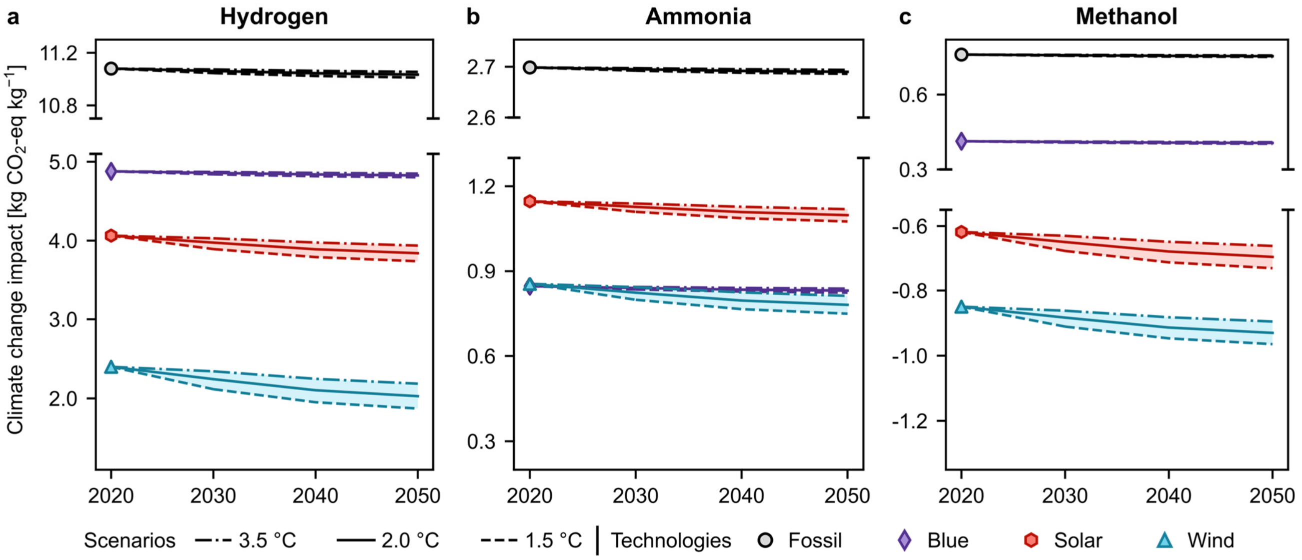

Fig. S5 Global average cradle-to-gate climate change impacts for fossil, blue, and green (both solar- and wind-based) production of **(a)** hydrogen, **(b)** ammonia, and **(c)** methanol from 2020 to 2050 under three climate policy scenarios, considering decarbonisation only in the materials sector such as cement and steel.

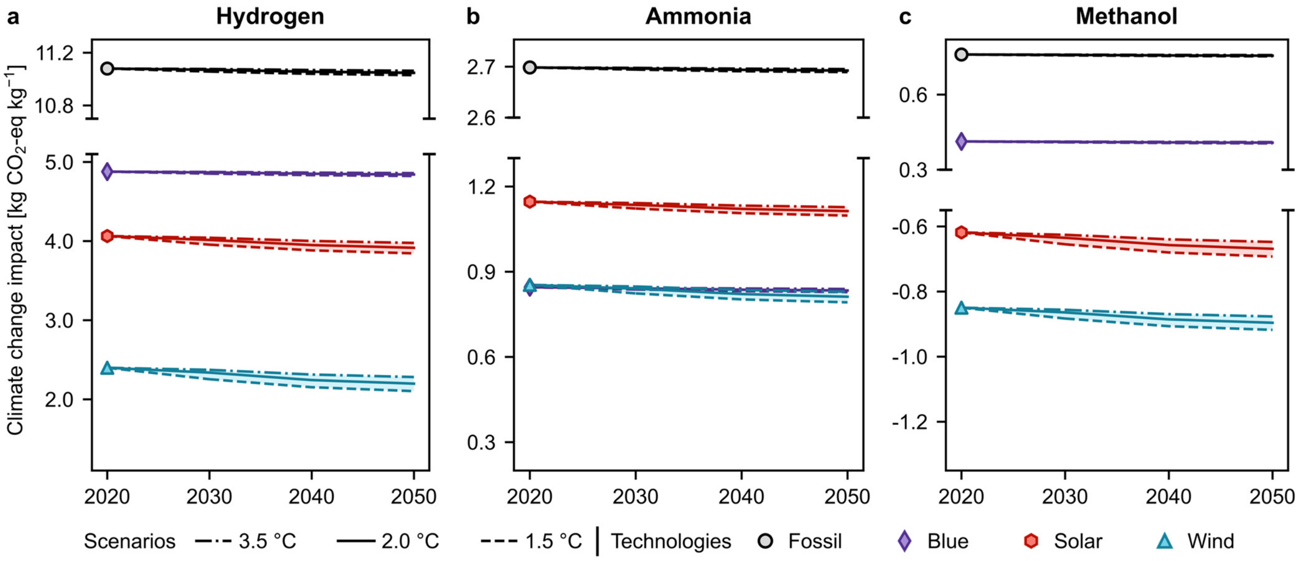

Fig. S6 Global average cradle-to-gate climate change impacts for fossil, blue, and green (both solar- and wind-based) production of **(a)** hydrogen, **(b)** ammonia, and **(c)** methanol from 2020 to 2050 under three climate policy scenarios, considering decarbonisation only in the transport sector.

7. Prospective techno-economic assessment

Conducting a prospective techno-economic assessment is challenging due to the high uncertainty and volatility inherent in future energy prices, influenced by geopolitical factors and technological advancements. Moreover, it remains unclear how learning curves will affect emerging technologies. Nonetheless, following previous studies,[20,21] a techno-economic assessment, along with a sensitivity analysis, was conducted as an exploratory study on the prospective production costs of ammonia and methanol. This assessment utilised future prices of hydrogen from water electrolysis, carbon dioxide from DAC, natural gas, and electricity estimated elsewhere. Additionally, we performed a sensitivity analysis for all the routes, considering the uncertainty ranges for the prices.

The cost parameters used in the calculations are outlined in **Table S3**. Mass and energy flows were obtained from the sources listed in **Table S1**, while the results of the techno-economic assessment are displayed in **Fig. S7**. Specifically, the operating expenditure (OPEX) was calculated based on the aforementioned mass and energy flows in **Table S1**, while the capital expenditure (CAPEX) term was directly taken from the original sources. Notably, the CAPEX contributions for fossil and green ammonia were extracted from D'Angelo *et al.*[10] With 2020 as the reference year, the CAPEX contribution is estimated to be approximately 0.13 USD kg^{-1} and 0.08 USD kg^{-1} of ammonia for the fossil and green routes, respectively. Similarly, for fossil and green methanol, the CAPEX contributions stood at around 0.06 and 0.09 USD kg^{-1} of methanol for the fossil and green routes, respectively.[22] All CAPEX contributions have been adjusted to their respective years using the average historical inflation, where the inflation estimates from 2010 to 2020 were utilised. The sources and assumptions used to obtain the results in **Table S3** are explained below.

7.1. Natural gas and electricity prices

The Annual Energy Outlook from the U.S. Energy Information Administration[23] was utilised to obtain the average prices of natural gas and electricity from 2020 to 2050, as shown in **Table S3**. For the low and high prices of natural gas and electricity, we assume a 50% deviation from the average value. It should be noted that the natural gas and electricity prices are primarily based on those in the United States and are adopted here for a global context due to the lack of availability of regional data, which might not be applicable worldwide. We also acknowledge that, due to the unavailability of future projections, our estimates follow a conservative approach that omits potential peak prices due to geopolitical factors (*e.g.*, Europe's energy crisis), which could make green routes outperform their fossil counterparts economically speaking.[21]

7.2. Solar and wind-based hydrogen prices

The costs for solar and wind-based hydrogen for 2020 were extracted from Nabera *et al.*[21] assuming the CAPEX for the electrolyser to be around 1124 USD kW^{-1} and the renewables costs ranging from 35 to 65 USD MWh^{-1} for wind and 39 to 163 USD MWh^{-1} for solar, respectively.[10] Furthermore, for future hydrogen costs, we used the estimates of the Hydrogen Council (average, low, and high values),[24] as shown in **Table S3**, which consider the projected levelised cost of electricity from IRENA[25] and potential declines in the CAPEX of the electrolyser to 750, 500, and 250 USD kW^{-1} in 2030, 2040, and 2050 respectively.[24]

7.3. Nitrogen and carbon dioxide from DAC prices

The costs for nitrogen used in ammonia production are derived from D'Angelo *et al.*[10] by adjusting for the average historical inflation across all future projections, using inflation estimates from 2010 to 2020. The lower and upper values were calculated assuming a 50% range. As for the costs of carbon dioxide from DAC used in methanol production, we rely on optimistic future prices and associated variability from Young *et al.*,[26] assuming high uptake and 100% technological dominance for solvent-based DAC technology. Since there is insufficient data available for 2040 regarding DAC prices, we assume a linear trend between 2030 and 2050 to calculate the prices of carbon dioxide for 2040, which applies to the average as well as the low and high values.

Table S3 Cost parameters used in techno-economic assessment. The values in **bold** are calculated using the assumptions outlined earlier.

**Flow**	**2020**			**2030**			**2040**			**2050**		
	**Avg.**	**Low**	**High**	**Avg.**	**Low**	**High**	**Avg.**	**Low**	**High**	**Avg.**	**Low**	**High**
Natural gas, feedstock[23] [USD m^{-3}]	0.072	**0.036**	**0.108**	0.116	**0.058**	**0.174**	0.123	**0.062**	**0.185**	0.128	**0.064**	**0.192**
Natural gas, heating[23] [USD GJ^{-3}]	1.964	**0.982**	**2.945**	3.168	**1.584**	**4.752**	3.367	**1.684**	**5.051**	3.500	**1.750**	**5.250**
Grid electricity[23] [USD kWh^{-1}]	0.104	**0.052**	**0.156**	0.103	**0.052**	**0.155**	0.101	**0.051**	**0.152**	0.096	**0.048**	**0.144**
Solar-based hydrogen[21,24] [USD kg^{-1}]	9.220	8.340	15.900	**6.670**	4.060	9.280	**5.280**	3.240	7.320	**3.927**	2.533	5.320
Wind-based hydrogen[21,24] [USD kg^{-1}]	6.880	5.230	8.480	**5.750**	3.500	8.000	**4.950**	3.038	6.863	**3.500**	2.400	4.600
Nitrogen, gaseous[10] [USD kg^{-1}]	0.089	**0.045**	**0.134**	**0.097**	**0.048**	**0.145**	**0.105**	**0.052**	**0.157**	**0.113**	**0.057**	**0.170**
Carbon dioxide, from DAC[26] [USD kg^{-1}]	0.362	0.269	0.623	0.192	0.123	0.469	**0.158**	**0.108**	**0.431**	0.123	0.092	0.392

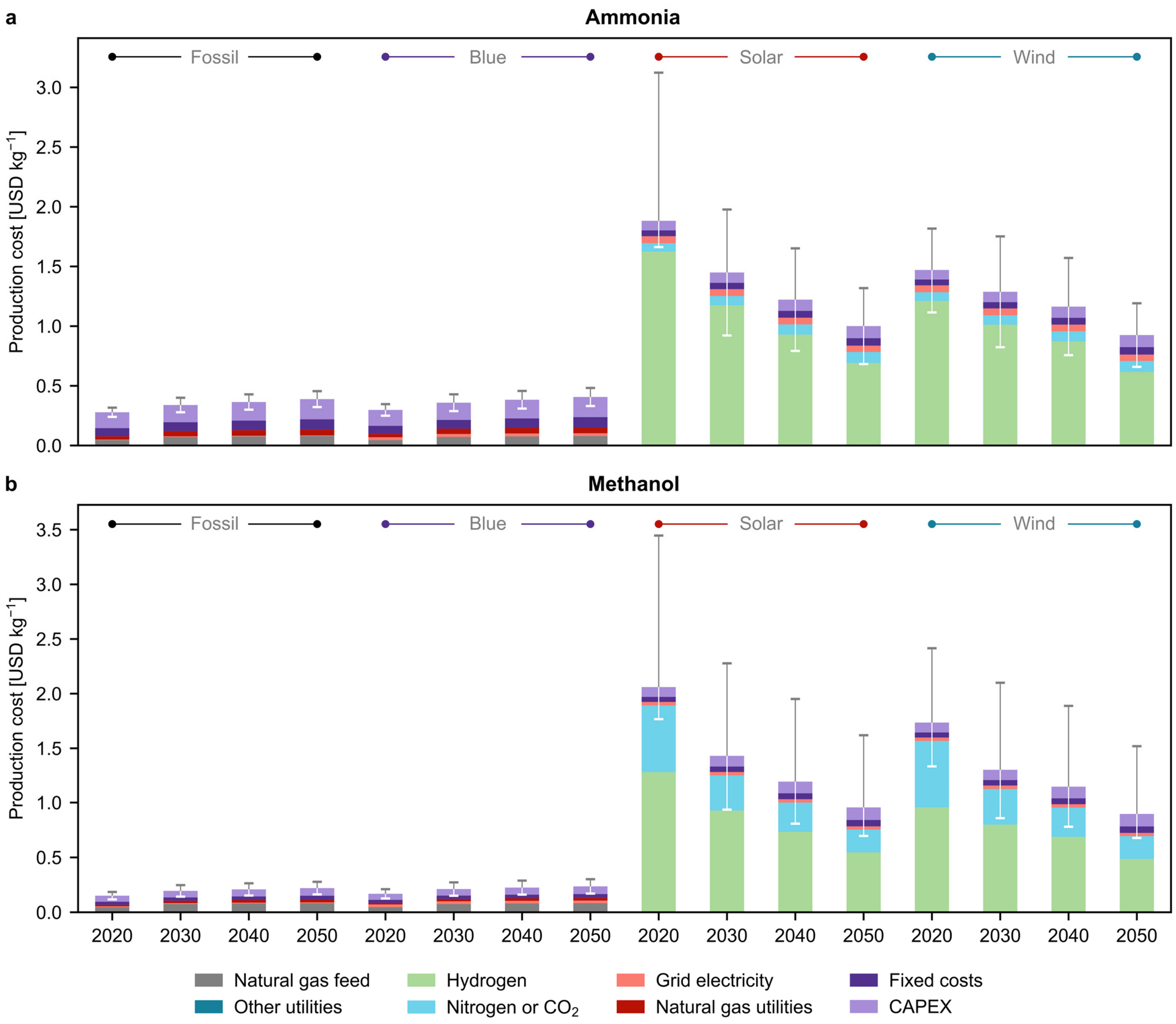

Fig. S7 Prospective techno-economic assessment for both **(a)** ammonia and **(b)** methanol production. The sensitivity analysis involved varying the prices of natural gas and grid electricity to calculate production cost estimates for both fossil and blue routes. For the green technologies, we used estimates for hydrogen, nitrogen and carbon dioxide prices. Error bars depict the production cost range of fossil and blue routes, utilising energy prices with low, average, and high values as shown in **Table S3**. The error bars for the green scenarios result from variations in the expected costs of hydrogen, nitrogen (for ammonia), or carbon dioxide from DAC (for methanol). This analysis is based on future projections from the literature and provides rough estimates due to the high uncertainty and volatility inherent in future energy prices, influenced by geopolitical factors and technological advancements. It is intended to be an exploratory analysis rather than to offer accurate estimates, which are hard to predict considering future uncertainties in global supply chains.

8. Ozone depletion and particulate matter formation impacts under all scenarios

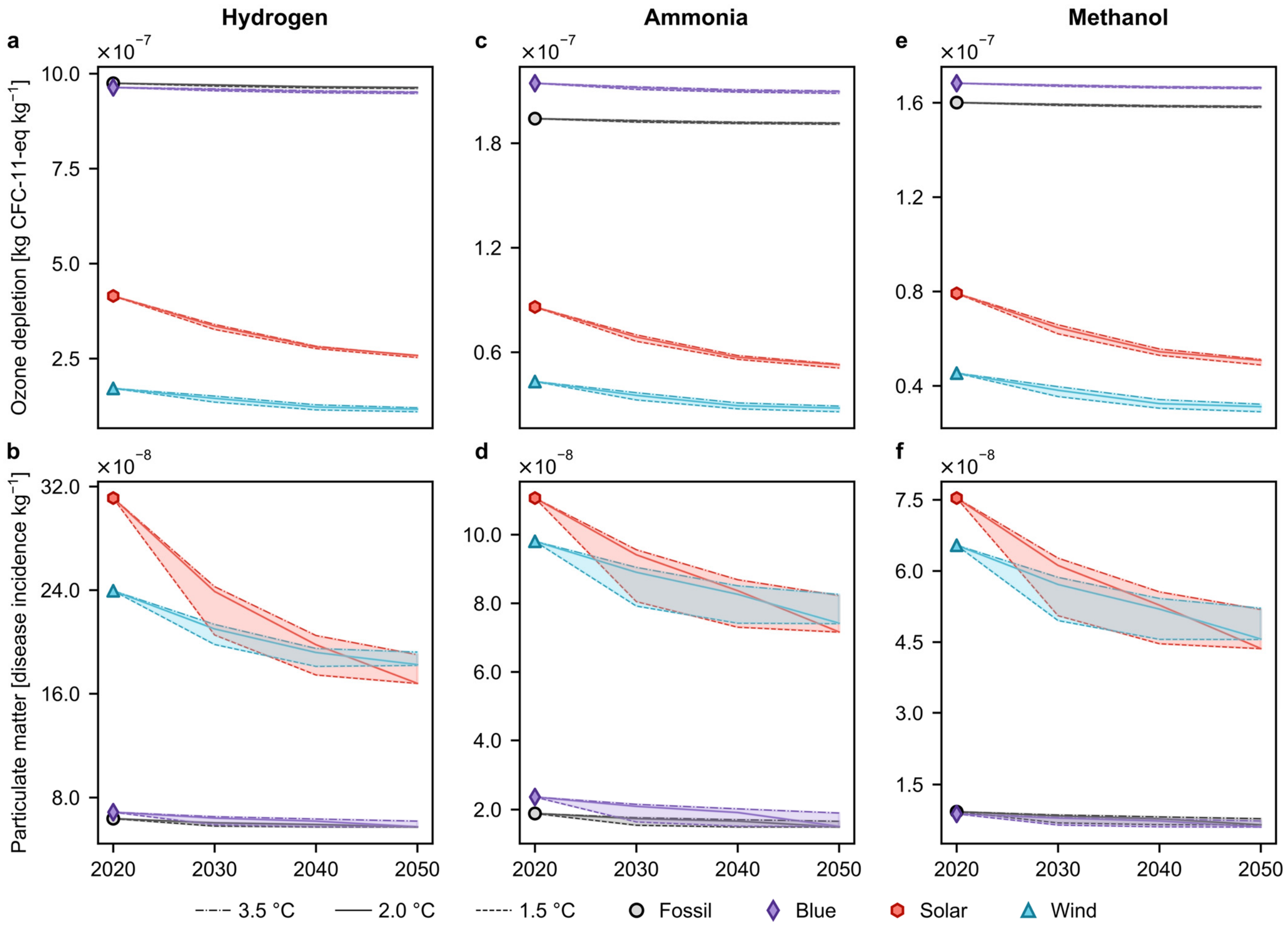

Fig. S8 Global average cradle-to-gate ozone depletion and particulate matter impacts of fossil, blue, and green (both solar- and wind-based) production of **(a)-(b)** hydrogen, **(c)-(d)** ammonia, and **(e)-(f)** methanol, from 2020 to 2050. These impact categories were selected based on their quality levels, following the recommendations of the European Commission's Joint Research Centre. Specifically, quality level I impact categories. The results reported are for the scenario compatible with limiting global warming below 3.5 °C, 2 °C, and 1.5 °C.

9. Breakdown of particulate matter and ozone depletion under the 2 °C scenario

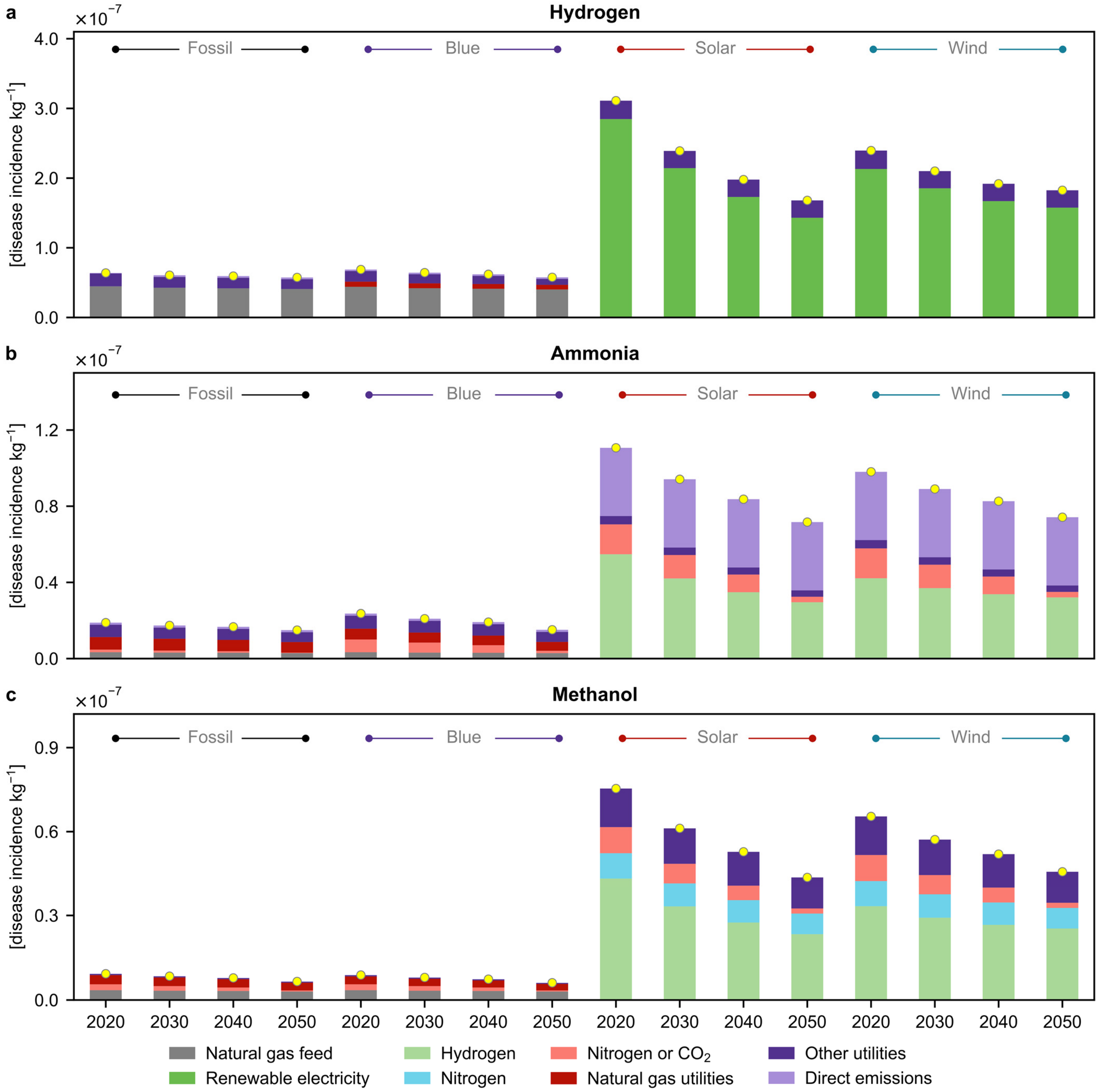

Fig. S9 Breakdown projections of global average cradle-to-gate particulate matter formation impacts for fossil, blue, and green (both solar- and wind-based) **(a)** hydrogen, **(b)** ammonia, and **(c)** methanol. The results reported are for the scenario compatible with limiting global warming below 2 °C. The yellow dots represent the net impact of the specified technology. In this category, we observe burden shifting for the green routes.

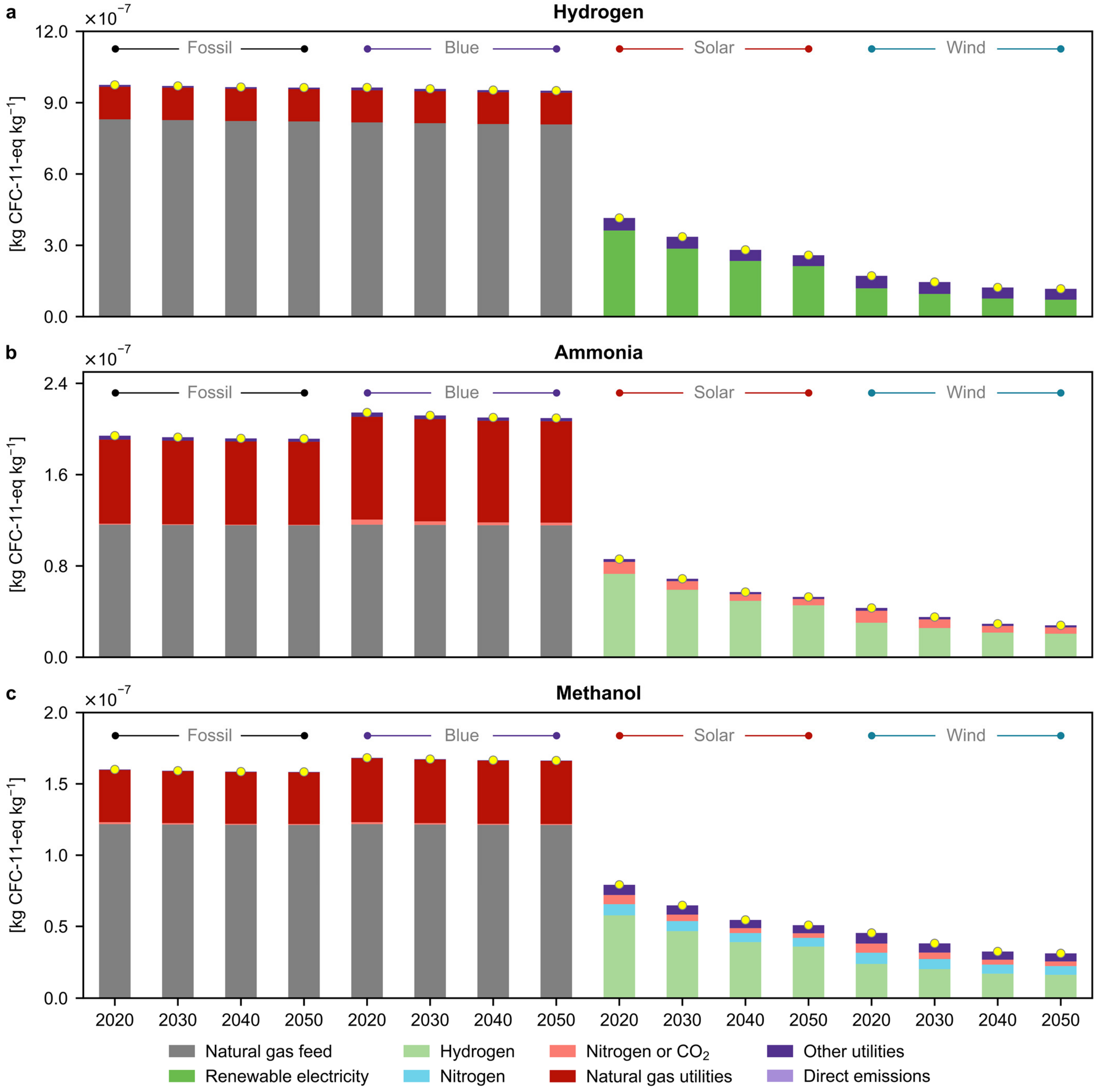

Fig. S10 Breakdown projections of global average cradle-to-gate ozone depletion impacts for fossil, blue, and green (both solar- and wind-based) **(a)** hydrogen, **(b)** ammonia, and **(c)** methanol. The results reported are for the scenario compatible with limiting global warming below 2 °C. The yellow dots represent the net impact of the specified technology. In general, fossil and blue routes display only marginal improvements.

10. Additional impact categories under the 2 °C scenario

As defined by the European Commission's Joint Research Centre, the main manuscript discusses quality level I (*i.e.*, satisfactory) impact categories, namely, climate change, ozone depletion, and particulate matter. Herein, we focus on 12 other impact categories belonging to quality level II (*i.e.*, some improvements needed) and quality level III (*i.e.*, apply with caution). All assessed impact categories are briefly described in **Table S4.**

Table S4 Brief description of other impact categories assessed in this study belonging to quality levels II and III.

**Category**	**Description**	**Quality level**	**Unit**
Acidification	Indicator of soil and water acidification potential, resulting from emission of nitrogen and sulphur oxides	II	kg mol H^+
Ecotoxicity freshwater	Effect of toxic substances on freshwater organisms	II	CTUe
Eutrophication: freshwater	Indicator of freshwater ecosystem enrichment with nutritional elements, resulting from emission of nitrogen or phosphorous containing compounds	II	kg PO_4-eq
Eutrophication: marine	Indicator of marine ecosystem enrichment with nutritional elements due to the emissions of nitrogen containing compounds	II	kg N-eq
Eutrophication: terrestrial	Indicator of terrestrial ecosystem enrichment with nutritional elements due to emission of nitrogen containing compounds	II	mol N-eq
Human toxicity: carcinogenic	Impact of toxic substances emitted on humans, categorised as cancer-related toxic substances	II	CTUh
Human toxicity: non-carcinogenic	Impact of toxic substances emitted on humans, categorised as non-cancer-related toxic substances	II	CTUh
Ionising radiation	Damage to human health and ecosystems caused by the emissions of radionuclides	II	kBq U-235
Photochemical ozone formation	Indicator of emissions that contribute to the formation of photochemical ozone in the lower atmosphere under the influence of sunlight	II	kg NMVOC-eq
Resource use: fossils	Indicator of the depletion of natural fossil fuel resources	III	MJ, net calorific value
Resource use: minerals and metals	Indicator of the depletion of natural non-fossil resources	III	kg Sb-eq
Water use	Indicator of the relative water usage derived from regionalised water scarcity factors	III	m^3 world eq. deprived

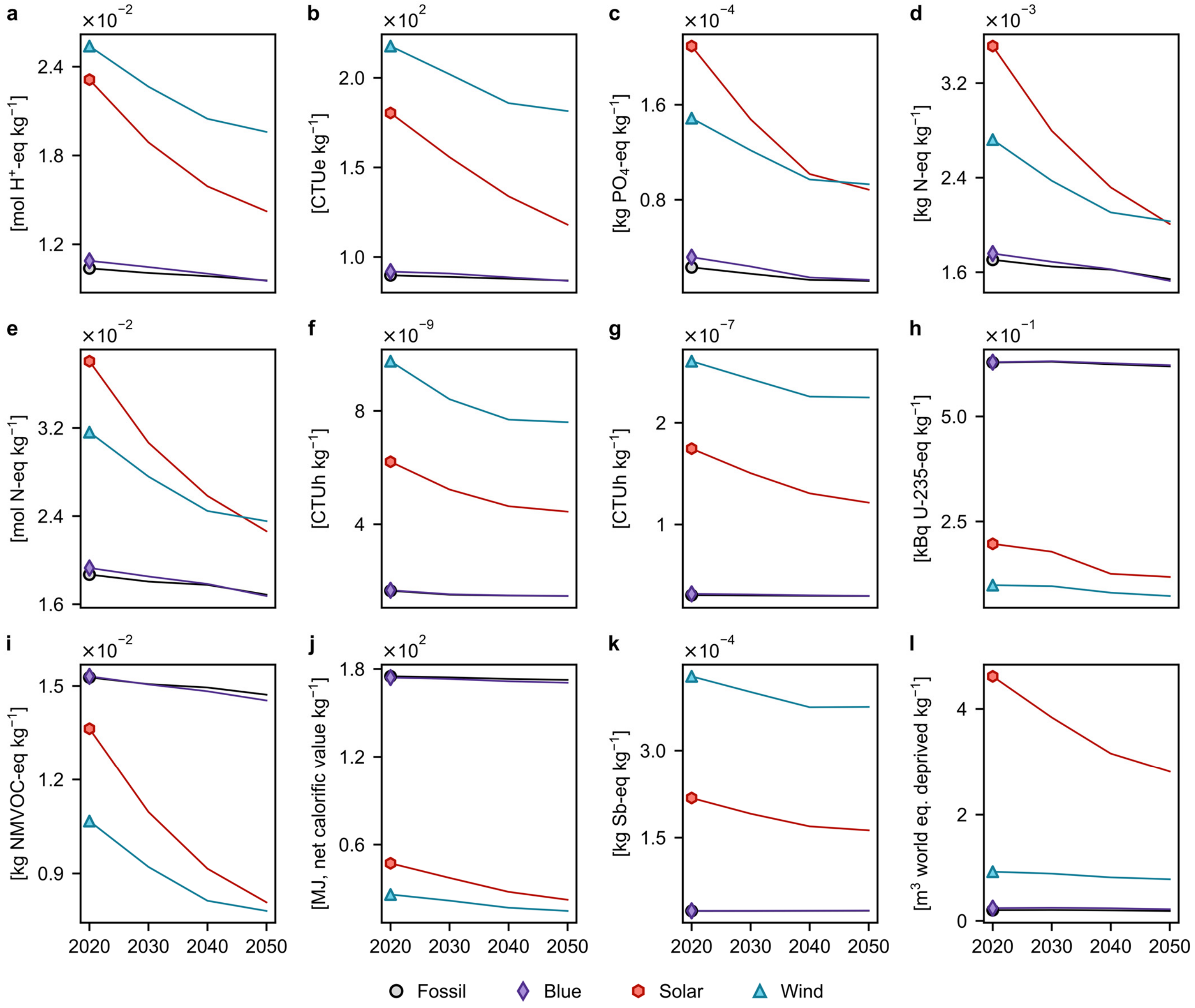

Fig. S11 Other environmental impacts of hydrogen, namely, **(a)** acidification, **(b)** ecotoxicity freshwater, **(c)** eutrophication freshwater, **(d)** eutrophication marine, **(e)** eutrophication terrestrial, **(f)** human toxicity carcinogenic, **(g)** human toxicity non-carcinogenic, **(h)** ionising radiation, **(i)** photochemical ozone formation, **(j)** resource use fossils, **(k)** resource use mineral and metals, and **(l)** water use per kg of hydrogen for different production technologies. The results reported are for the scenario compatible with limiting global warming below 2 °C.

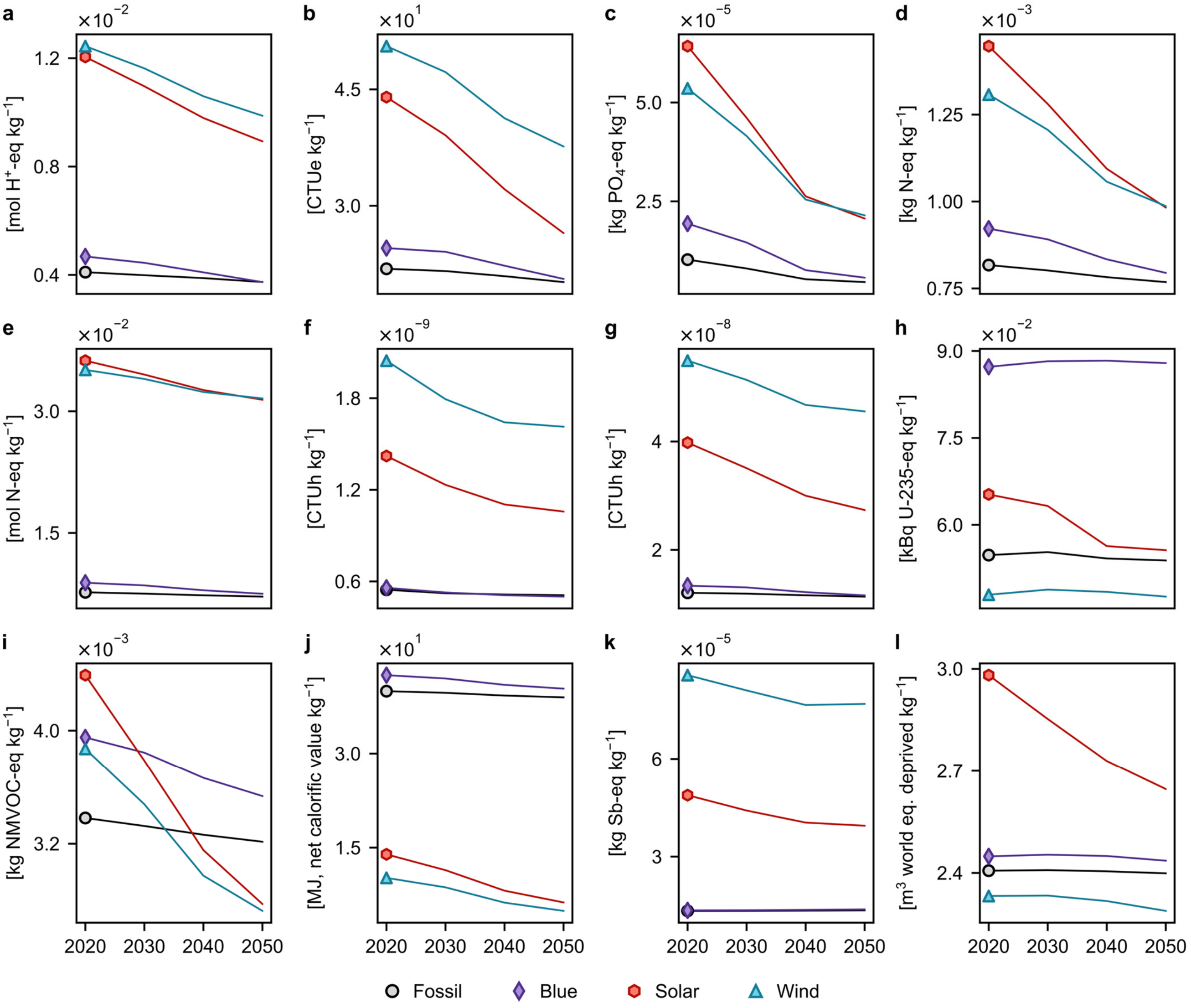

Fig. S12 Other environmental impacts of ammonia, namely, **(a)** acidification, **(b)** ecotoxicity freshwater, **(c)** eutrophication freshwater, **(d)** eutrophication marine, **(e)** eutrophication terrestrial, **(f)** human toxicity carcinogenic, **(g)** human toxicity non-carcinogenic, **(h)** ionising radiation, **(i)** photochemical ozone formation, **(j)** resource use fossils, **(k)** resource use mineral and metals, and **(l)** water use per kg of ammonia for different production technologies. The results reported are for the scenario compatible with limiting global warming below 2 °C.

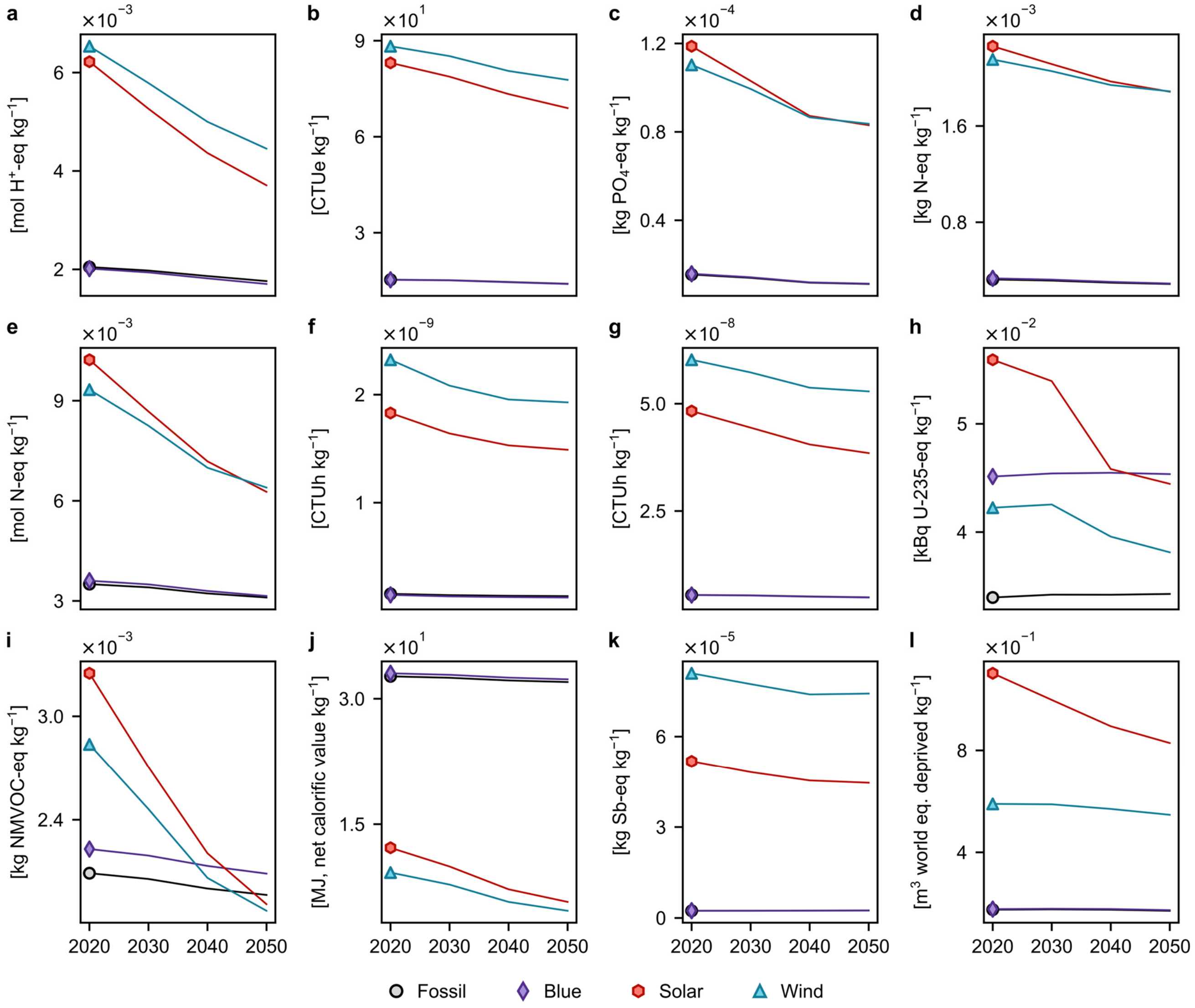

Fig. S13 Other environmental impacts of methanol, namely, **(a)** acidification, **(b)** ecotoxicity freshwater, **(c)** eutrophication freshwater, **(d)** eutrophication marine, **(e)** eutrophication terrestrial, **(f)** human toxicity carcinogenic, **(g)** human toxicity non-carcinogenic, **(h)** ionising radiation, **(i)** photochemical ozone formation, **(j)** resource use fossils, **(k)** resource use mineral and metals, and **(l)** water use per kg of methanol for different production technologies. The results reported are for the scenario compatible with limiting global warming below 2 °C.

11. Additional regional implementation roadmaps

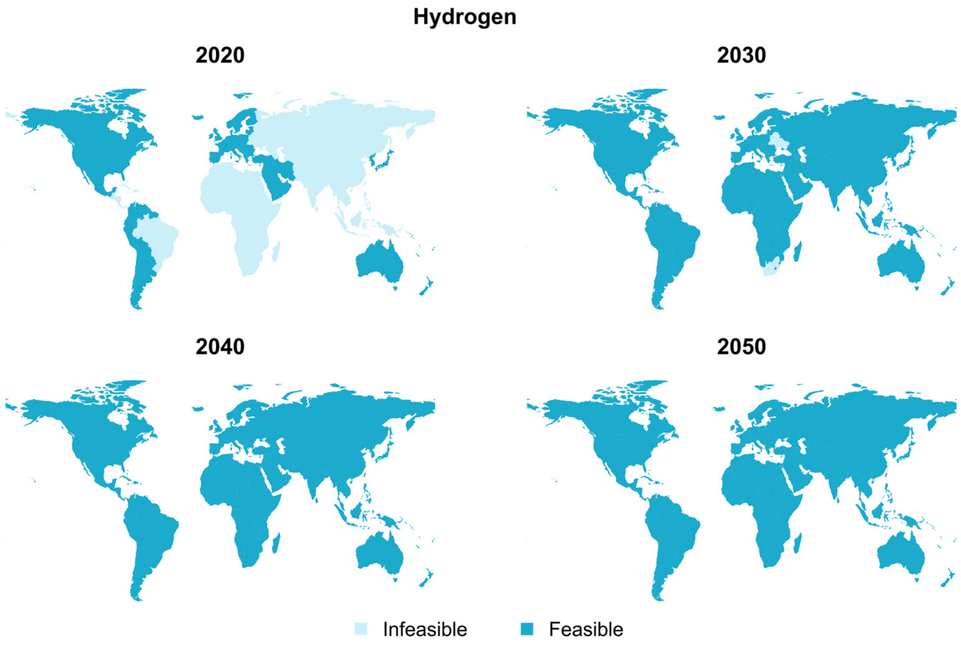

Fig. S14 Regional assessment of the cradle-to-gate climate change impacts for wind-based hydrogen. The highlighted regions in blue for a specific year indicate locations with impacts lower than a defined threshold of 80% reduction from their respective fossil-based impacts in 2020 (8.9 kg CO_2-eq kg^{-1}) considering scenarios compatible with limiting global warming below 2 °C.

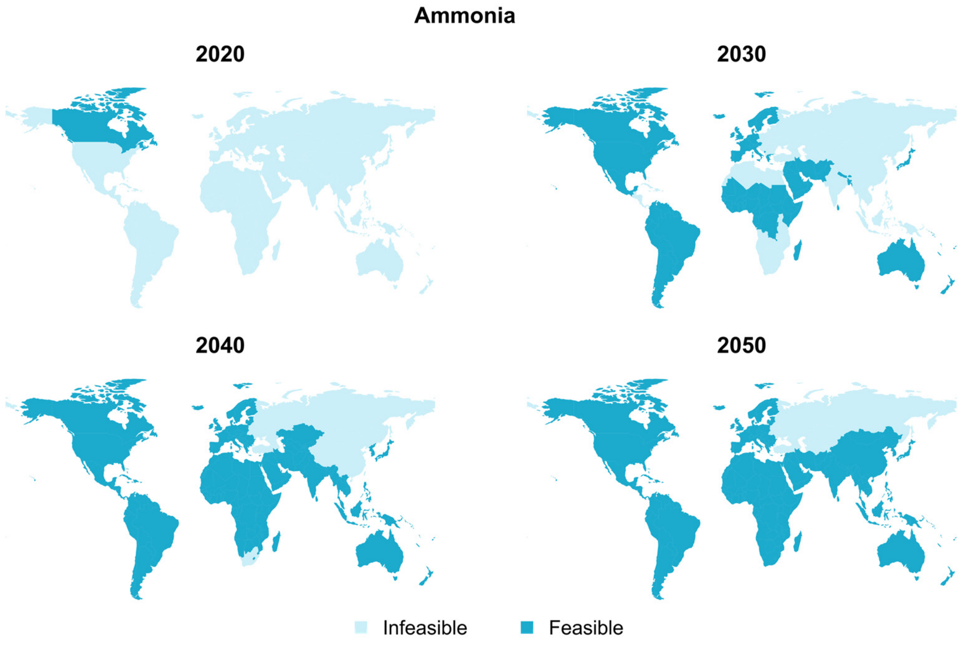

Fig. S15 Regional assessment of the cradle-to-gate climate change impacts for wind-based ammonia. The highlighted regions in blue for a specific year indicate locations with impacts lower than a defined threshold of 80% reduction from their respective fossil-based impacts in 2020 (2.2 kg CO_2-eq kg^{-1}) considering scenarios compatible with limiting global warming below 2 °C.

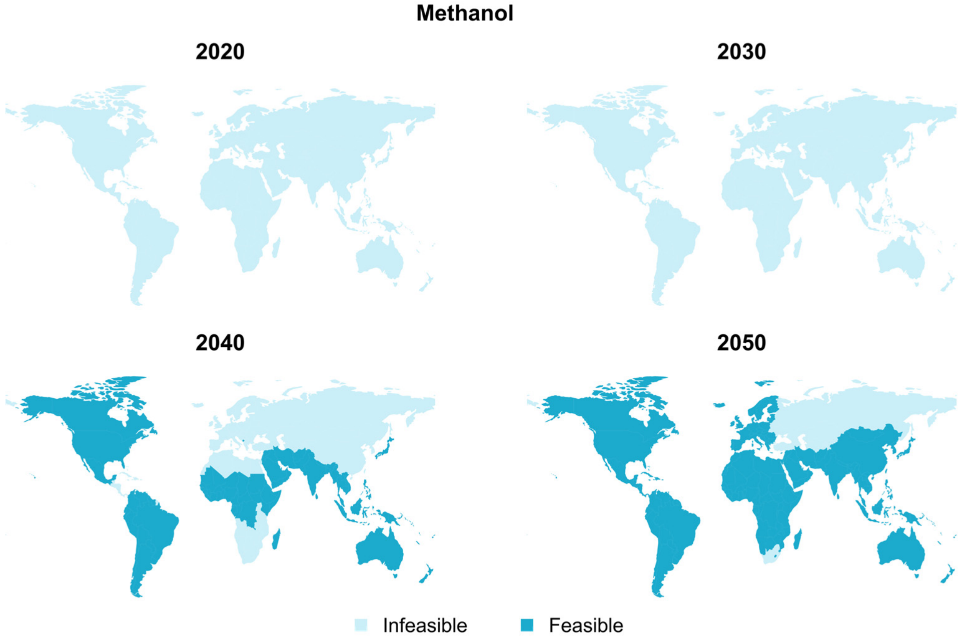

Fig. S16 Regional assessment of the cradle-to-gate climate change impacts for wind-based production routes of methanol. The highlighted regions in blue for a specific year indicate locations with impacts lower than a defined threshold of a 50% reduction from their respective wind-based impacts in 2020 (–1.2 kg CO_2-eq kg^{-1}), considering scenarios compatible with limiting global warming below 2 °C.

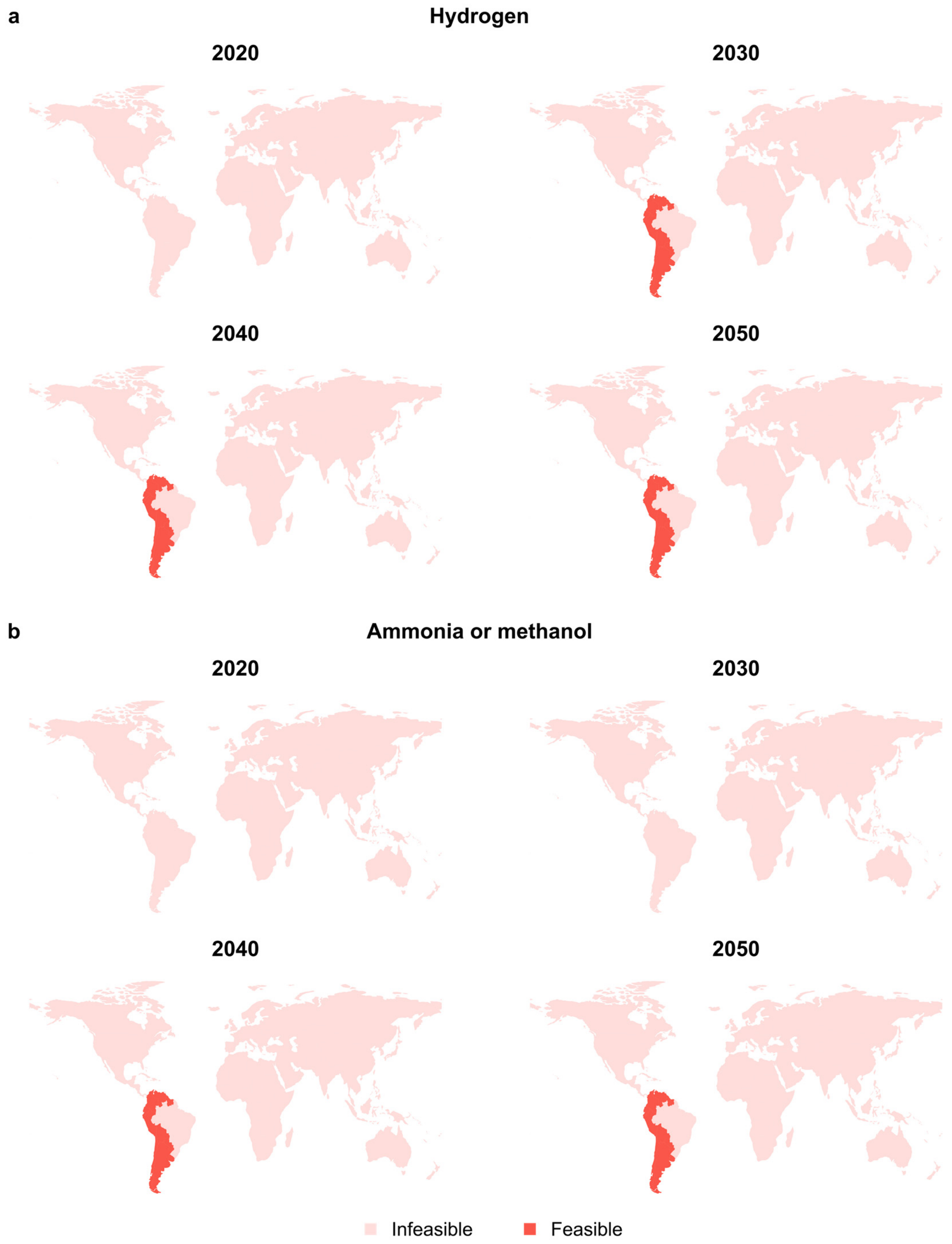

Fig. S17 Regional assessment of the cradle-to-gate climate change impacts for solar-based production routes of **(a)** hydrogen, **(b)** ammonia or methanol. The highlighted regions in red for a specific year indicate locations with impacts lower than a defined threshold, considering scenarios compatible with limiting global warming below 3.5 °C. For hydrogen and ammonia, thresholds of 80% reduction from their respective fossil-based impacts in 2020 (8.9 and 2.2 kg CO_2-eq kg^{-1}, respectively) are considered. Meanwhile, for methanol, an 80% reduction from its respective solar-based impacts in 2020 (–1.1 kg CO_2-eq kg^{-1}) is utilised due to its net negative impacts.

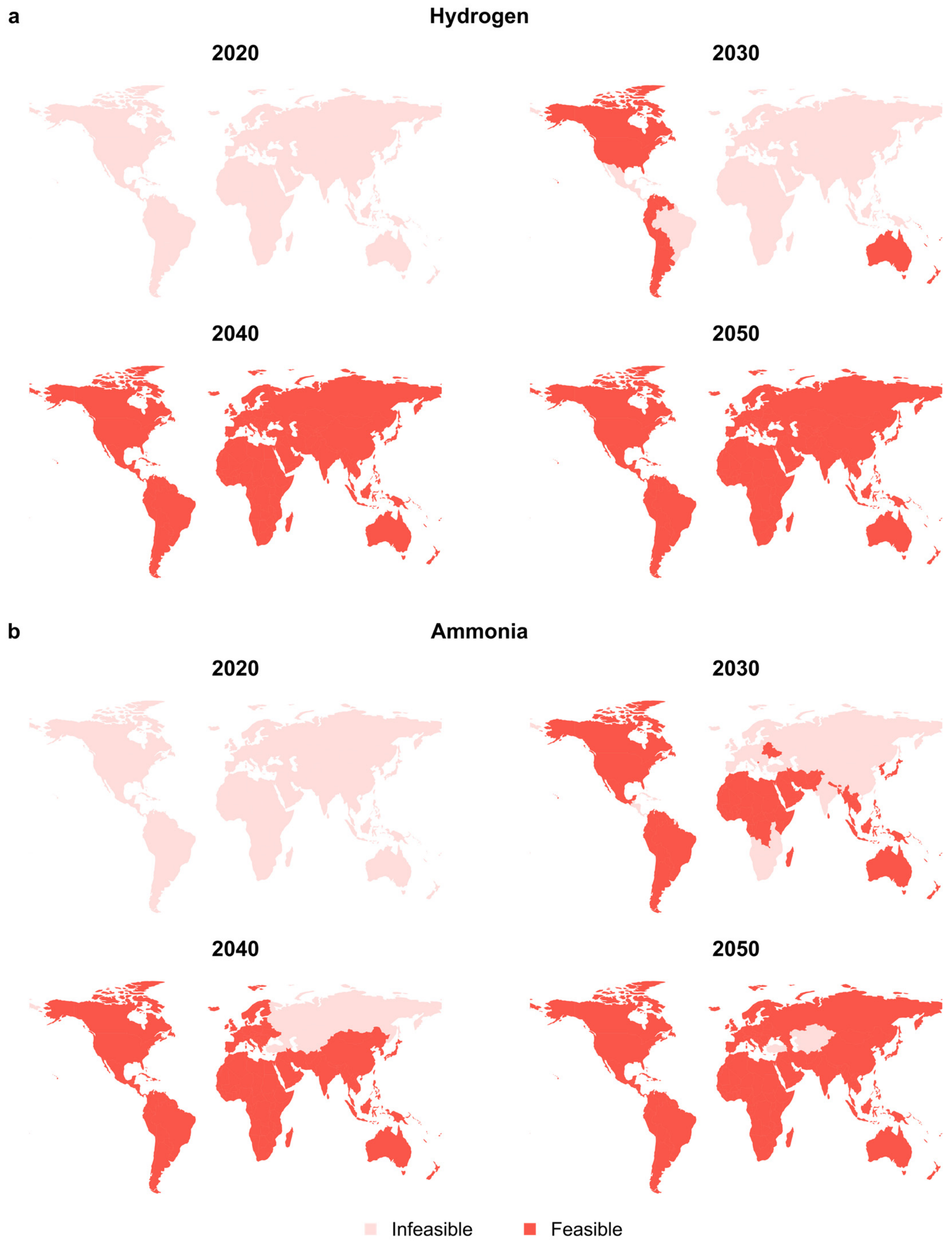

Fig. S18 Regional assessment of the cradle-to-gate climate change impacts for solar-based production routes of **(a)** hydrogen, **(b)** ammonia or methanol. The highlighted regions in red for a specific year indicate locations with impacts lower than a defined threshold, considering scenarios compatible with limiting global warming below 1.5 °C. For hydrogen and ammonia, thresholds of 80% reduction from their respective fossil-based impacts in 2020 (8.9 and 2.2 kg CO_2-eq kg^{-1}, respectively) are considered. Meanwhile, for methanol, an 80% reduction from its respective solar-based impacts in 2020 (–1.1 kg CO_2-eq kg^{-1}) is utilised due to its net negative impacts.

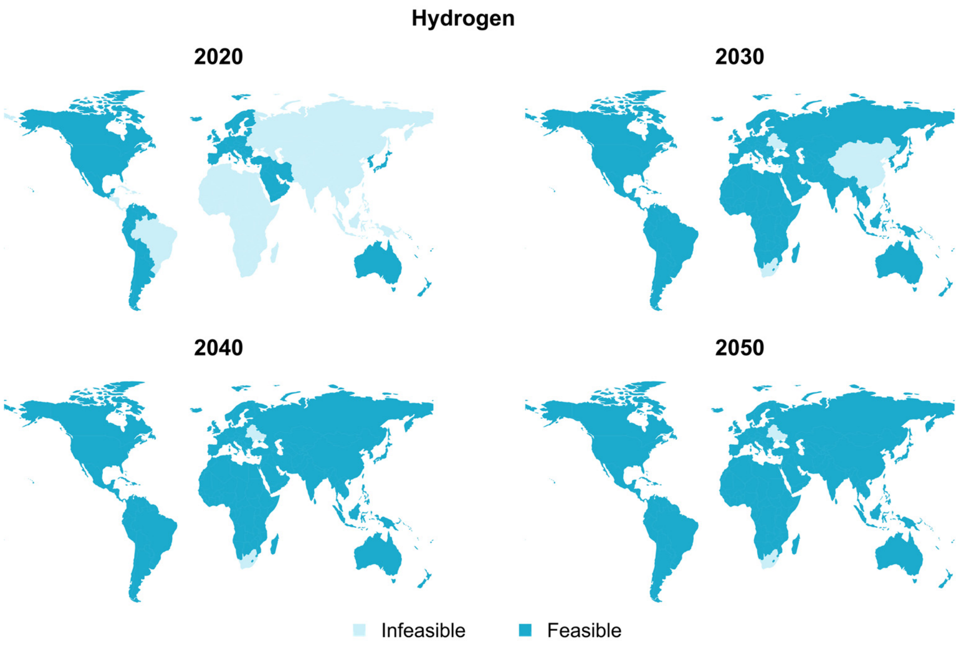

Fig. S19 Regional assessment of the cradle-to-gate climate change impacts for wind-based hydrogen. The highlighted regions in blue for a specific year indicate locations with impacts lower than a defined threshold of 80% reduction from their respective fossil-based impacts in 2020 (8.9 kg CO_2-eq kg^{-1}) considering scenarios compatible with limiting global warming below 3.5 °C.

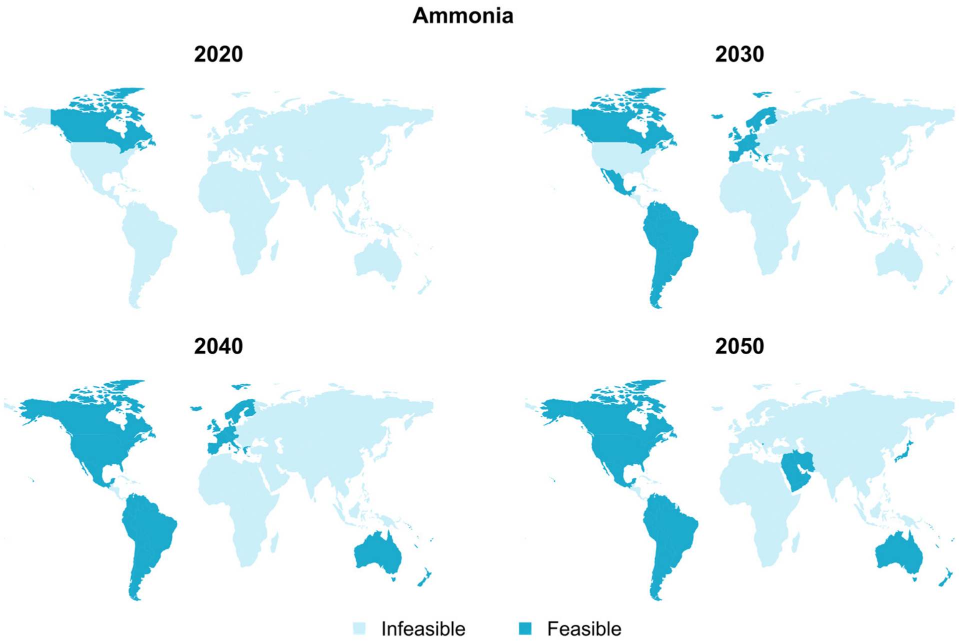

Fig. S20 Regional assessment of the cradle-to-gate climate change impacts for wind-based ammonia. The highlighted regions in blue for a specific year indicate locations with impacts lower than a defined threshold of 80% reduction from their respective fossil-based impacts in 2020 (2.2 kg CO_2-eq kg^{-1}) considering scenarios compatible with limiting global warming below 3.5°C.

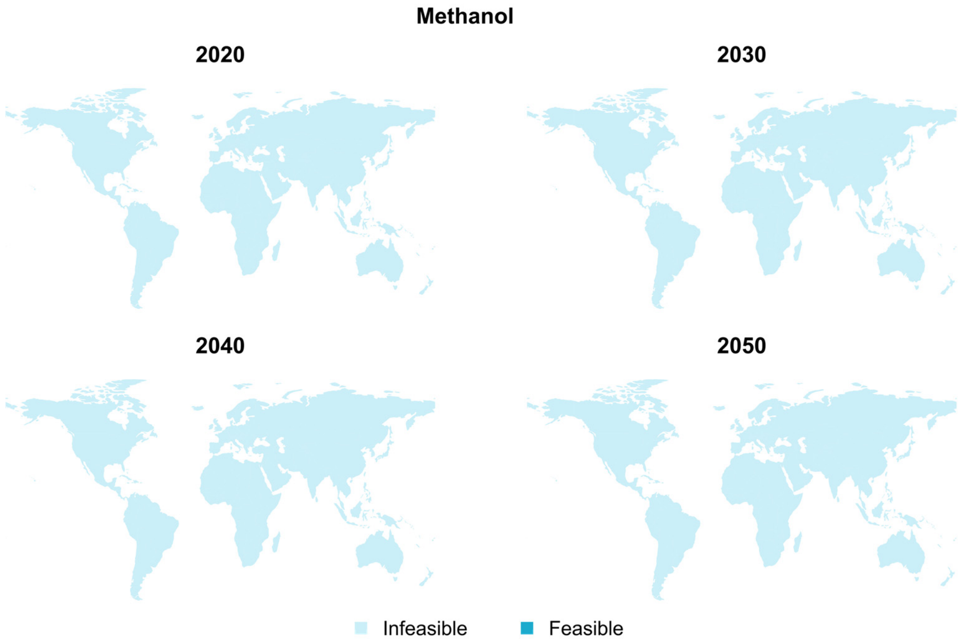

Fig. S21 Regional assessment of the cradle-to-gate climate change impacts for wind-based methanol. The highlighted regions in blue for a specific year indicate locations with impacts lower than a defined threshold of a 50% reduction from their respective wind-based impacts in 2020 (–1.2 kg CO_2-eq kg^{-1}), considering scenarios compatible with limiting global warming below 3.5 °C.

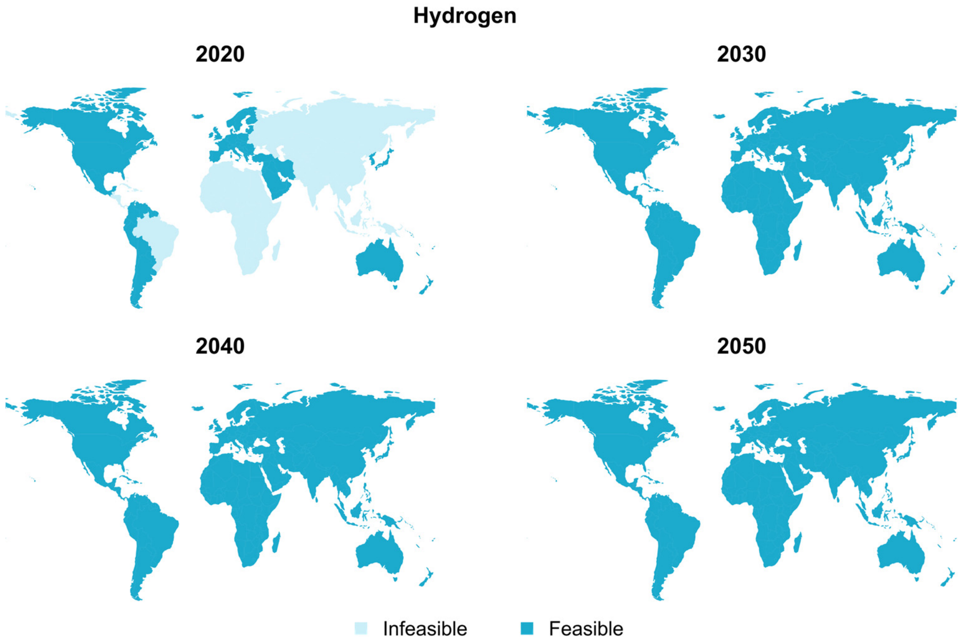

Fig. S22 Regional assessment of the cradle-to-gate climate change impacts for wind-based hydrogen. The highlighted regions in blue for a specific year indicate locations with impacts lower than a defined threshold of 80% reduction from their respective fossil-based impacts in 2020 (8.9 kg CO_2-eq kg^{-1}) considering scenarios compatible with limiting global warming below 1.5 °C.

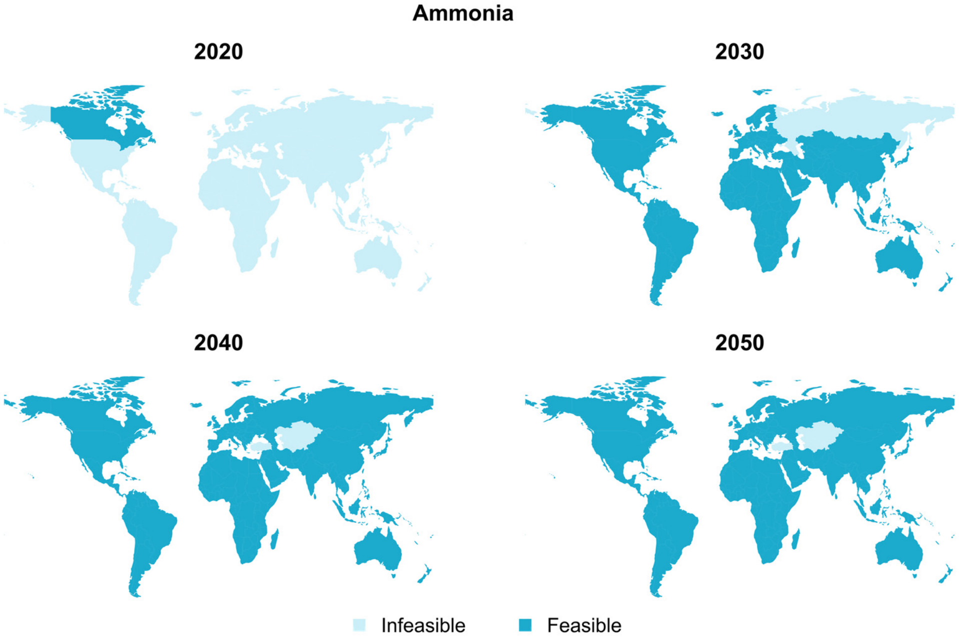

Fig. S23 Regional assessment of the cradle-to-gate climate change impacts for wind-based ammonia. The highlighted regions in blue for a specific year indicate locations with impacts lower than a defined threshold of 80% reduction from their respective fossil-based impacts in 2020 (2.2 kg CO_2-eq kg^{-1}) considering scenarios compatible with limiting global warming below 1.5 °C.

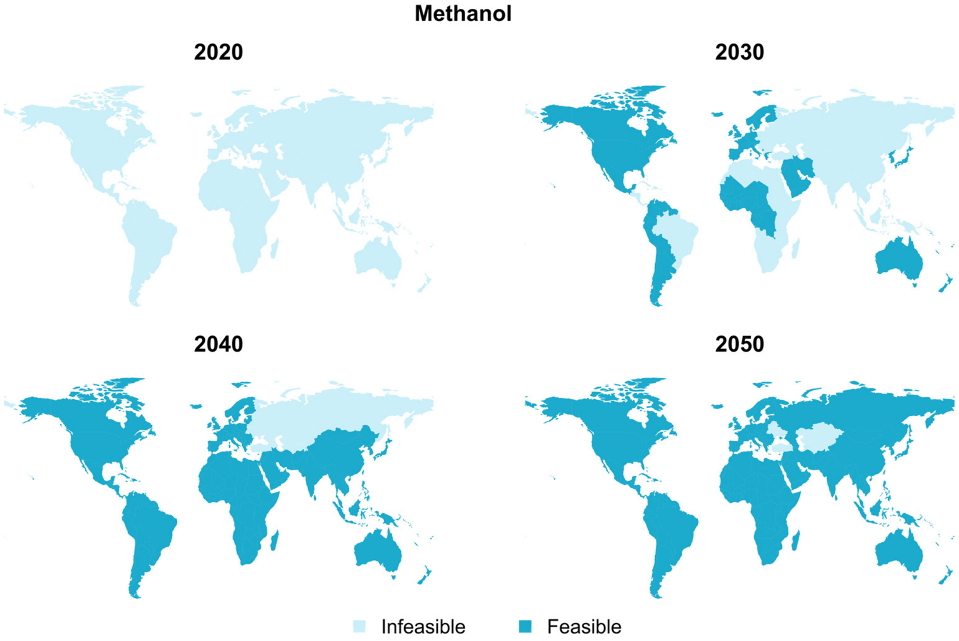

Fig. S24 Regional assessment of the cradle-to-gate climate change impacts for wind-based methanol. The highlighted regions in blue for a specific year indicate locations with impacts lower than a defined threshold of a 50% reduction from their respective wind-based impacts in 2020 (–1.2 kg CO_2-eq kg^{-1}), considering scenarios compatible with limiting global warming below 1.5 °C.

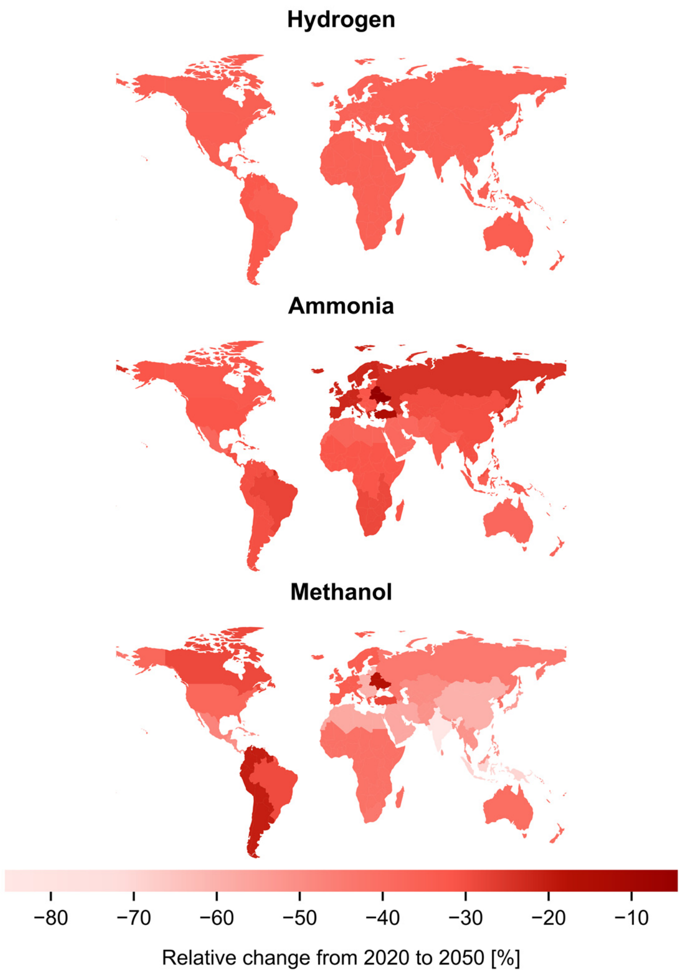

Fig. S25 Regional variability of the cradle-to-gate climate change impacts for solar-based production routes of hydrogen, ammonia, and methanol. The results indicate the percentage change for each specific region in 2050 relative to 2020, considering the scenario compatible with limiting global warming below 3.5 °C.

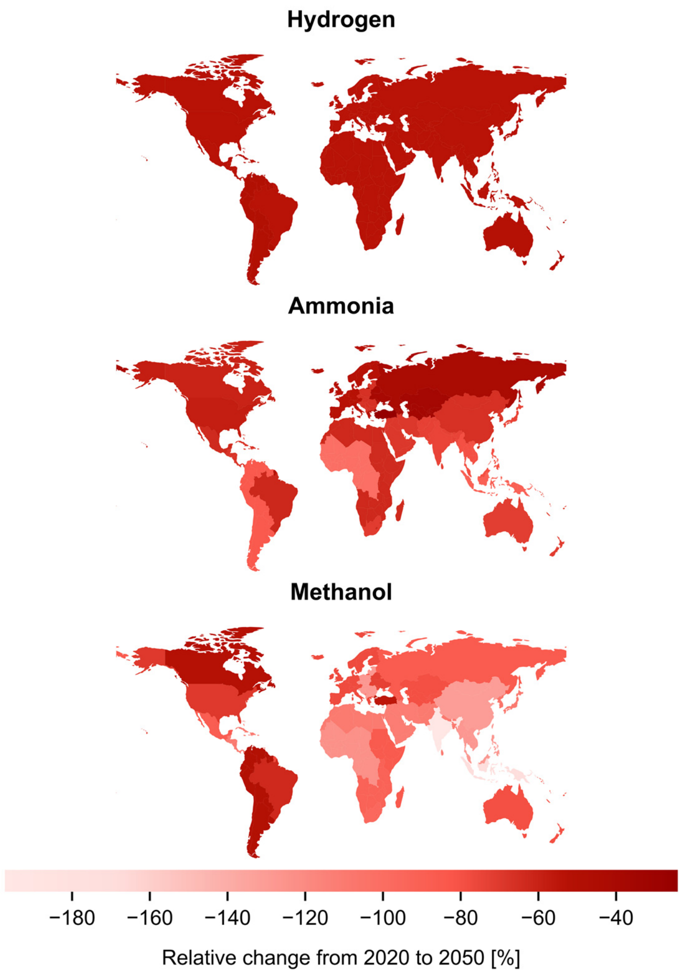

Fig. S26 Regional variability of the cradle-to-gate climate change impacts for solar-based production routes of hydrogen, ammonia, and methanol. The results indicate the percentage change for each specific region in 2050 relative to 2020, considering the scenario compatible with limiting global warming below 2 °C.

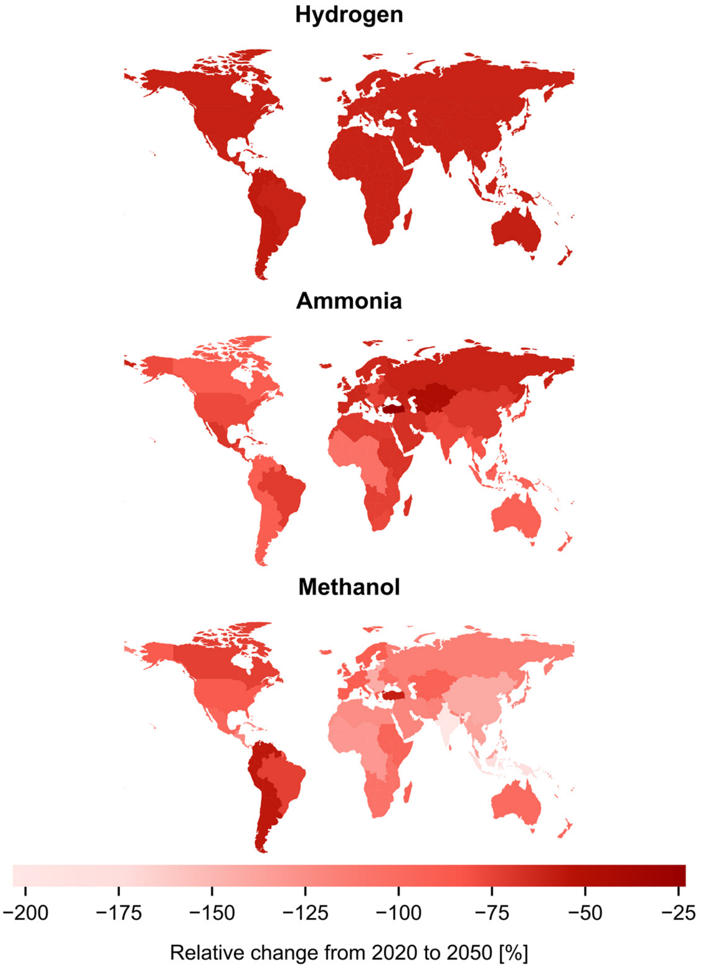

Fig. S27 Regional variability of the cradle-to-gate climate change impacts for solar-based production routes of hydrogen, ammonia, and methanol. The results indicate the percentage change for each specific region in 2050 relative to 2020, considering the scenario compatible with limiting global warming below 1.5 °C.

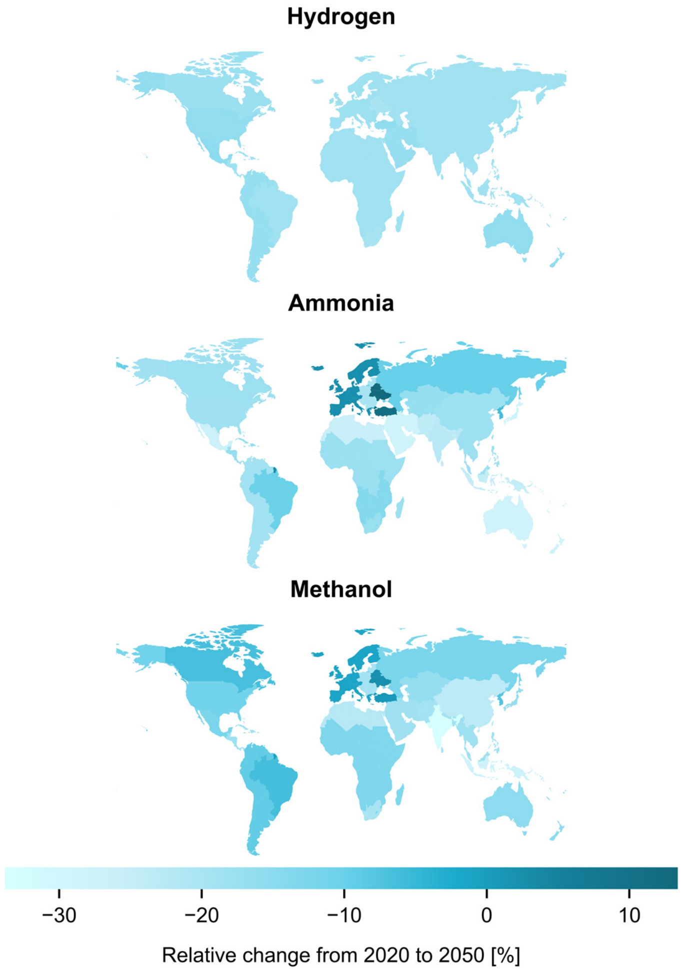

Fig. S28 Regional variability of the cradle-to-gate climate change impacts for onshore wind-based production routes of hydrogen, ammonia, and methanol. The results indicate the percentage change for each specific region in 2050 relative to 2020, considering the scenario compatible with limiting global warming below 3.5 °C.

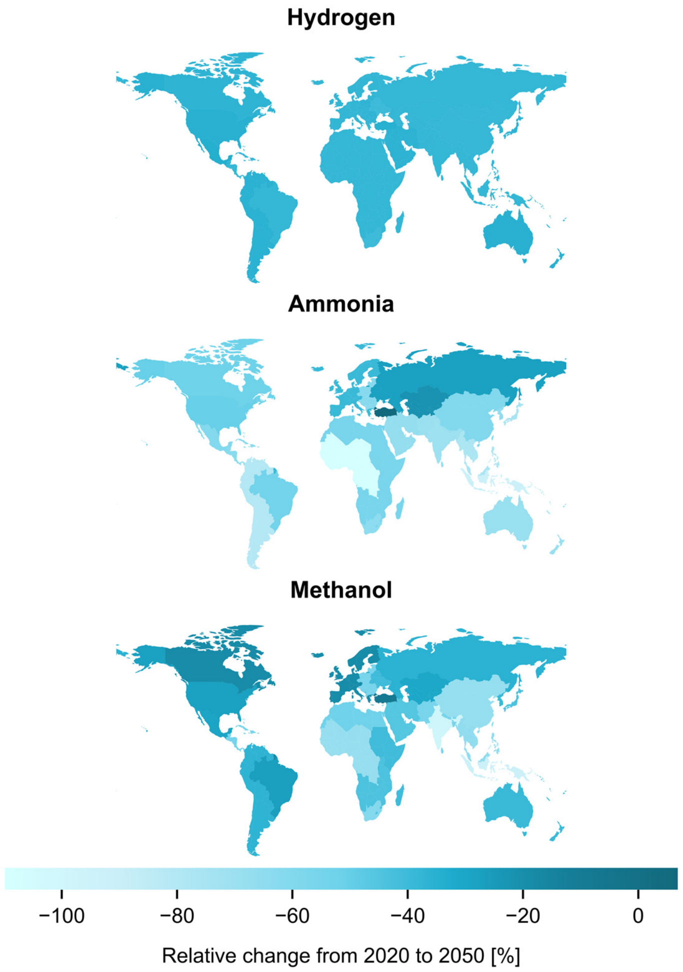

Fig. S29 Regional variability of the cradle-to-gate climate change impacts for onshore wind-based production routes of hydrogen, ammonia, and methanol. The results indicate the percentage change for each specific region in 2050 relative to 2020, considering the scenario compatible with limiting global warming below 2 °C.

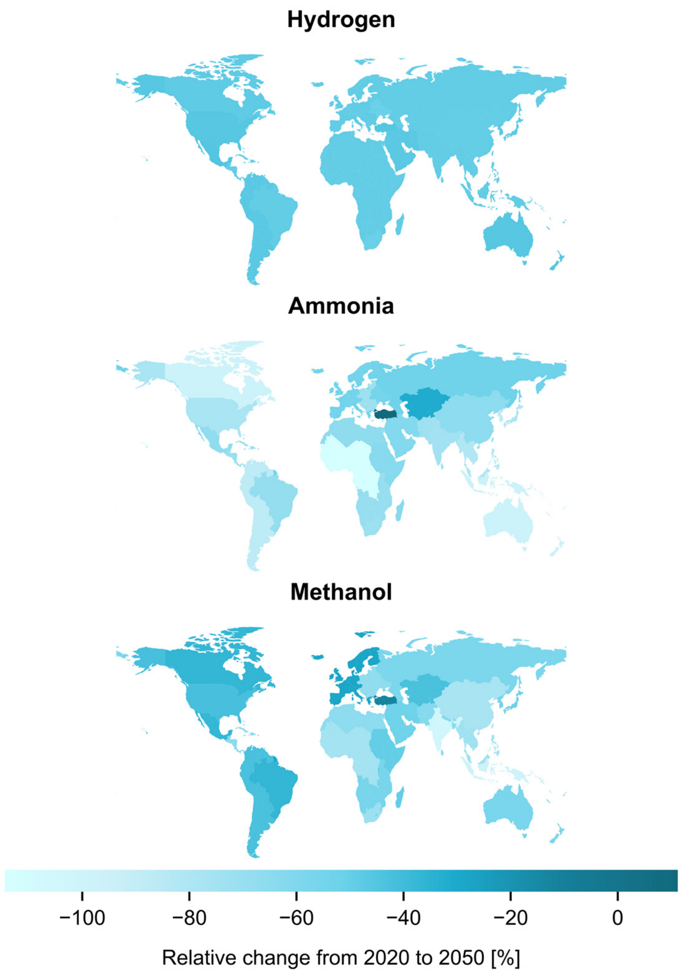

Fig. S30 Regional variability of the cradle-to-gate climate change impacts for onshore wind-based production routes of hydrogen, ammonia, and methanol. The results indicate the percentage change for each specific region in 2050 relative to 2020, considering the scenario compatible with limiting global warming below 1.5 °C.

References

1 T. Terlouw, C. Bauer, R. McKenna and M. Mazzotti, *Energy Environ. Sci.*, 2022, **15**, 3583–3602.

2 B. Parkinson, P. Balcombe, J. F. Speirs, A. D. Hawkes and K. Hellgardt, *Energy Environ. Sci.*, 2019, **12**, 19–40.

3 International Standards Organization, *In ISO 14040:2006 Environmental Management--Life Cycle Assessment--Principles and Framework*, 2006.

4 International Standards Organization, *In ISO 14044:2006 Environmental Management--Life Cycle Assessment--Requirements and Guidelines*, 2006.

5 V. Tulus, J. Pérez-Ramírez and G. Guillén-Gosálbez, *Green Chem.*, 2021, **23**, 9881–9893.

6 R. Sacchi, T. Terlouw, K. Siala, A. Dirnaichner, C. Bauer, B. Cox, C. Mutel, V. Daioglou and G. Luderer, *Renew. Sustain. Energy Rev.*, 2022, **160**, 112311.

7 G. Wernet, C. Bauer, B. Steubing, J. Reinhard, E. Moreno-Ruiz and B. Weidema, *Int. J. Life Cycle Assess.*, 2016, **21**, 1218–1230.

8 C. Antonini, K. Treyer, A. Streb, M. Van Der Spek, C. Bauer and M. Mazzotti, *Sustain. Energy Fuels*, 2020, **4**, 2967–2986.

9 K. Bareiß, C. De La Rua, M. Möckl and T. Hamacher, *Appl. Energy*, 2019, **237**, 862–872.

10 S. C. D’Angelo, S. Cobo, V. Tulus, A. Nabera, A. J. Martín, J. Pérez-Ramírez and G. Guillén-Gosálbez, *ACS Sustain. Chem. Eng.*, 2021, **9**, 9740–9749.

11 C. Hank, L. Lazar, F. Mantei, M. Ouda, R. J. White, T. Smolinka, A. Schaadt, C. Hebling and H.-M. Henning, *Sustain. Energy Fuels*, 2019, **3**, 3219–3233.

12 *Integrated Assessment of Global Environmental Change with IMAGE 3.0*, PBL Netherlands Environmental Assessment Agency, 2014.

13 C. Mutel, *J. Open Source Softw.*, 2017, **2**, 236.

14 C. B. Field and V. R. Barros, *Climate change 2014: impacts, adaptation, and vulnerability Working Group II contribution to the fifth assessment report of the Intergovernmental panel on climate change*, Cambridge university press, New York, 2014.

15 F. Biganzoli, L. Ceriani, E. Crenna, R. Pant, S. Sala, E. Saouter, D. Versteeg and L. Zampori, *Environmental footprint: update of Life Cycle Impact Assessment Methods : ecotoxicity freshwater, human toxicity cancer, and non-cancer*, Publications Office of the European Union, Luxembourg, 2018.

16 R. Hiederer, *International reference life cycle data system (ILCD) handbook: general guide for life cycle assessment: provisions and action steps*, Publications Office, Luxembourg, First edition., 2011.

17 K. Volkart, C. Bauer and C. Boulet, *Int. J. Greenh. Gas Control*, 2013, **16**, 91–106.

18 Y. Qiu, P. Lamers, V. Daioglou, N. McQueen, H.-S. De Boer, M. Harmsen, J. Wilcox, A. Bardow and S. Suh, *Nat. Commun.*, 2022, **13**, 3635.

19 R. Besseau, R. Sacchi, I. Blanc and P. Pérez-López, *Renew. Sustain. Energy Rev.*, 2019, **108**, 274–288.

20 I. Ioannou, S. C. D’Angelo, A. J. Martín, J. Pérez-Ramírez and G. Guillén-Gosálbez, *ChemSusChem*, 2020, **13**, 6370–6380.

21 A. Nabera, I.-R. Istrate, A. J. Martín, J. Pérez-Ramírez and G. Guillén-Gosálbez, *Green Chem.*, 2023, **25**, 6603–6611.

22 A. González-Garay, M. S. Frei, A. Al-Qahtani, C. Mondelli, G. Guillén-Gosálbez and J. Pérez-Ramírez, *Energy Environ. Sci.*, 2019, **12**, 3425–3436.

23 U.S. Energy Information Administration, Annual Energy Outlook 2021.

24 Hydrogen Council, Path to Hydrogen Competitiveness: A Cost Perspective.

25 *Global renewables outlook: energy transformation 2050*, International Renewable Energy Agency, Abu Dhabi, Energy transformation 2050., 2020.

26 J. Young, N. McQueen, C. Charalambous, S. Foteinis, O. Hawrot, M. Ojeda, H. Pilorgé, J. Andresen, P. Psarras, P. Renforth, S. Garcia and M. Van Der Spek, *One Earth*, 2023, **6**, 899–917.
